# MicroRNA-15b regulates mitochondrial ROS production and the senescence-associated secretory phenotype through sirtuin 4/SIRT4

**DOI:** 10.18632/aging.100905

**Published:** 2016-02-26

**Authors:** Alexander Lang, Susanne Grether-Beck, Madhurendra Singh, Fabian Kuck, Sascha Jakob, Andreas Kefalas, Simone Altinoluk-Hambüchen, Nina Graffmann, Maren Schneider, Antje Lindecke, Heidi Brenden, Ingo Felsner, Hakima Ezzahoini, Alessandra Marini, Sandra Weinhold, Andrea Vierkötter, Julia Tigges, Stephan Schmidt, Kai Stühler, Karl Köhrer, Markus Uhrberg, Judith Haendeler, Jean Krutmann, Roland P. Piekorz

**Affiliations:** ^1^ Institut für Biochemie und Molekularbiologie II, Universitätsklinikum der Heinrich-Heine-Universität, Düsseldorf, Germany; ^2^ IUF – Leibniz Research Institute for Environmental Medicine, Düsseldorf, Germany; ^3^ Institut für Transplantationsdiagnostik und Zelltherapeutika (ITZ), Düsseldorf, Germany; ^4^ Biologisch-Medizinisches Forschungszentrum (BMFZ), Düsseldorf, Germany; ^5^ Molecular Proteomics Laboratory, BMFZ, Universitätsklinikum der Heinrich-Heine-Universität, Düsseldorf, Germany; ^6^ University of Düsseldorf, Medical Faculty, Düsseldorf, Germany

**Keywords:** MicroRNA-15b/miR-15b, nuclear encoded mitochondrial genes, photoaging, reactive oxygen species/ROS, senescence, senescence associated secretory phenotype/SASP, SIRT4/Sirtuin 4, skin

## Abstract

Mammalian sirtuins are involved in the control of metabolism and life-span regulation. Here, we link the mitochondrial sirtuin SIRT4 with cellular senescence, skin aging, and mitochondrial dysfunction. SIRT4 expression significantly increased in human dermal fibroblasts undergoing replicative or stress-induced senescence triggered by UVB or gamma-irradiation. *In-vivo*, SIRT4 mRNA levels were upregulated in photoaged *vs.* non-photoaged human skin. Interestingly, in all models of cellular senescence and in photoaged skin, upregulation of SIRT4 expression was associated with decreased levels of miR-15b. The latter was causally linked to increased SIRT4 expression because miR-15b targets a functional binding site in the *SIRT4* gene and transfection of oligonucleotides mimicking miR-15b function prevented SIRT4 upregulation in senescent cells. Importantly, increased SIRT4 negatively impacted on mitochondrial functions and contributed to the development of a senescent phenotype. Accordingly, we observed that inhibition of miR-15b, in a SIRT4-dependent manner, increased generation of mitochondrial reactive oxygen species, decreased mitochondrial membrane potential, and modulated mRNA levels of nuclear encoded mitochondrial genes and components of the senescence-associated secretory phenotype (SASP). Thus, miR-15b is a negative regulator of stress-induced SIRT4 expression thereby counteracting senescence associated mitochondrial dysfunction and regulating the SASP and possibly organ aging, such as photoaging of human skin.

## INTRODUCTION

Extrinsic skin aging (*i.e*., photoaging) is primarily driven by solar ultraviolet (UV) radiation as a major environmental noxae [1]. The exact mechanisms, by which UV radiation lead to skin aging, is an area of active investigation. Interestingly, it has been shown that in addition to UV radiation-induced stress responses in the epidermal compartment, which e.g. may elicit the production and release of soluble factors like proteolytic enzymes, damage to dermal fibroblasts contributes to photoaging as well [2]. Accordingly, mitochondrial dysfunction in dermal fibroblasts associated with increased production of reactive oxygen species (“defective powerhouse model”) has been proposed as a major driving force in extrinsic aging of human skin (reviewed in [3]). This concept is supported by the observation of Koziel et al. that in skin fibroblasts from aged donors the mitochondrial membrane potential is significantly decreased, whereas levels of reactive oxygen species (ROS) are increased [4], a major hallmark of fibroblast aging [5].

In general, these findings are in line with the idea that DNA damage triggered by UV radiation or other extrinsic factors, including oxidative stress or cytotoxic drugs, causes cellular aging referred to as stress-induced premature senescence (SIPS) [6]. This phenotype is typically associated with and dependent on mitochondrial dysfunction [7-11]. In addition, human cells display an intrinsic type of senescence (replicative senescence) which is based on the progressive erosion of telomeres. Senescent cells are characterized by several features which distinguish them from quiescent cells reversibly arrested in the G_1_ phase of the cell cycle [6, 12]. In particular, upon senescence, cells undergo morphological changes such as flattening and increased granularity, formation of senescence associated heterochromatic foci (SAHF) linked to the repression of proliferative genes and hence DNA synthesis, and increased senescence associated β-galactosidase (SA-βgal) expression based on an expansion of the lysosomal compartment [13, 14]. A particular characteristic of senescent cells is their ability to produce and secrete various autocrine- or paracrine-acting factors, including cytokines, growth factors, proteases, and soluble receptors, giving rise to the so-called senescence-associated secretory phenotype (SASP) [14-18]. We recently demonstrated that premature senescence can also be induced as a consequence of mitotic spindle stress elicited by cellular depletion of the centrosomal protein TACC3, a major regulator of mitotic spindle dynamics [19-21]. In this model system, centrosomal and spindle dysfunction were found to modulate post-mitotic senescence through a p53-p21^WAF^ dependent pathway [22].

In the present study we investigated the causes for mitochondrial dysfunction in cellular senescence in general [10] and fibroblast senescence and photoaging of human skin in particular [3, 4] by focusing on sirtuins. Mammalian sirtuins comprise a conserved protein family of seven members of NAD^+^-dependent deacetylases or, in the case of SIRT4, ADP-ribosyltransferases [23, 24], which are all expressed in human skin [25]. The prototype sirtuin, SIRT1, plays diverse and senescence/aging-associated roles in metabolism and stress biology. Downregulation or inhibition of SIRT1 is associated with metabolic dysregulation and antagonization of anti-aging effects linked to calorie restriction, impaired DNA repair, and apoptosis in the context of cellular stress responses [26-28]. Moreover, SIRT1 contributes to telomere maintenance [29] and is downregulated during cellular senescence [30]. In contrast to all other sirtuins that localize in the cytosol or nucleus, SIRT3, SIRT4, and SIRT5 are exclusively found in the mitochondrium, an organelle linking energy metabolism/metabolic homeostasis and aging [31-34]. Important metabolic enzymes, like glutamate dehydrogenase (GDH) and pyruvate dehydrogenase (PDH) in the case of SIRT4, have been identified as specific substrates of mitochondrial SIRT proteins (mtSIRTs) [23, 35-37], but the regulation and putative roles of mtSIRTs, in particular SIRT4, in intrinsically and extrinsically induced cellular senescence and tissue aging are currently poorly understood.

## RESULTS AND DISCUSSION

### SIRT4 is upregulated in various models of cellular senescence

**Figure 1:**
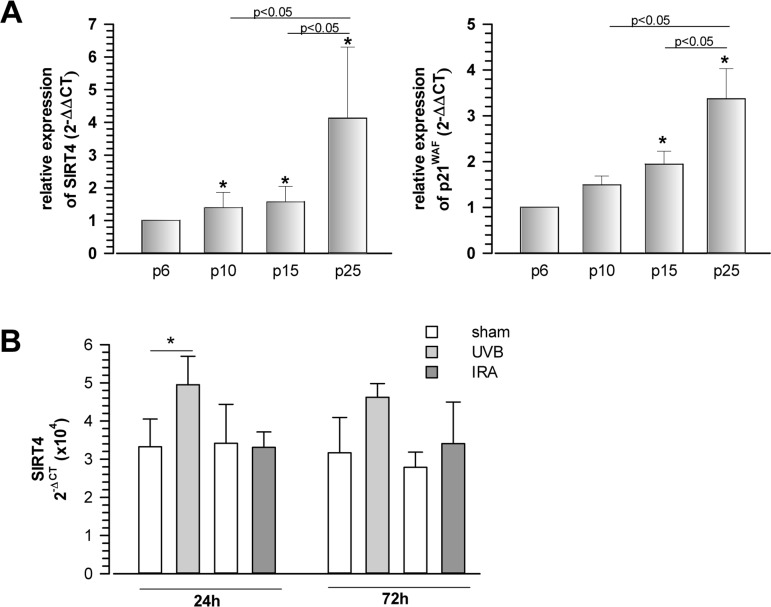
Increased expression of the mitochondrial sirtuin SIRT4 in human fibroblasts undergoing replicative or UVB-induced senescence (**A**) Concomitant upregulation of SIRT4 and p21^WAF^ expression during replicative senescence. Human foreskin fibroblast lines (from n=4 donors; mean ± s.d.) were analysed at increasing passage numbers for SIRT4 and p21^WAF^ mRNA levels by quantitative real-time polymerase chain reaction (qRT-PCR; suppl. Material & Methods). To evaluate statistical significance, ANOVA (on ranks for SIRT4) SNK were performed (*p<0.05). (**B**) Primary human dermal fibroblasts (n=4 donors; mean ± s.d.) were subjected to repetitive ultraviolet irradiation (100 J/m^2^ UVB per day) or infrared irradiation (360 J/m^2^ UVB per day) for a total of five days. Cells were harvested 24 and 72 h following the last irradiation and SIRT4 mRNA levels were determined by qRT-PCR. To evaluate statistical significance ANOVA on ranks (SNK) were performed (*p<0.05).

In the present study, we addressed a possible role of mtSIRTs in aging by analyzing their expression during cellular senescence and tissue aging. Initially, we observed that SIRT4 mRNA levels were upregulated at later passages during replicative senescence of human foreskin fibroblasts, comparable to the parallel increase in expression of the p53-induced cell cycle inhibitor and senescence effector p21^WAF^ (Fig. 1A). The increase in SIRT4 mRNA levels was not specific to replicative senescence, as SIRT4 transcripts were found at elevated levels in primary human dermal fibroblasts, in which senescence was induced extrinsically by repetitive exposure to low, sublethal doses of ultraviolet radiation (UV) B radiation [38, 39] (Fig. 1B).

**Figure 2:**
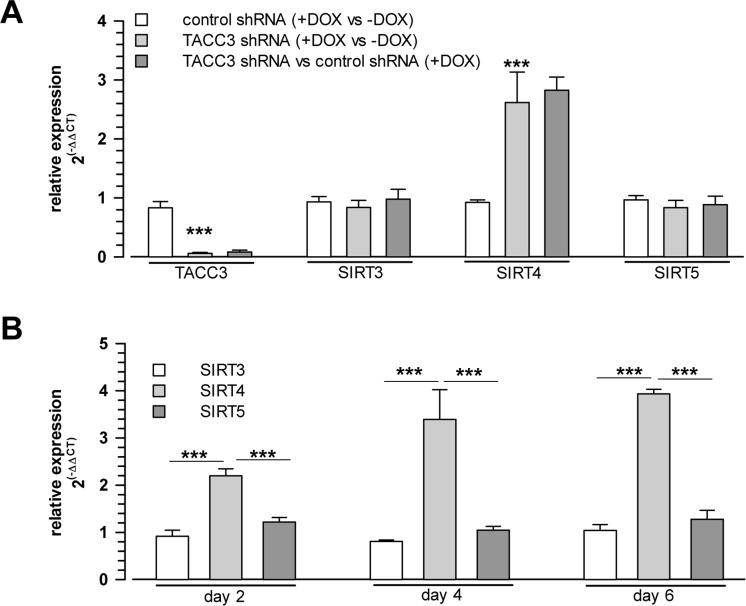
Selective upregulation of SIRT4 expression among the mitochondrial SIRT isoforms in cellular models of stress-induced premature senescence (**A**) Increased SIRT4 mRNA levels in MCF7 cells driven into premature senescence through shRNA-mediated depletion of the centrosomal protein TACC3 [22] (analysis on day 4 upon doxycycline treatment which induces control or TACC3 shRNA expression; mean ± s.d. from three independent experiments). (**B**) Upregulation of SIRT4 mRNA levels in MCF7 cells 2, 4 and 6 days following γ-irradiation (single dose of 20 Gy; mean ± s.d. from three independent experiments). mRNA levels were determined by quantitative real-time polymerase chain reaction. To evaluate statistical significance, ANOVA SNK were performed (***p<0.001).

**Figure 3:**
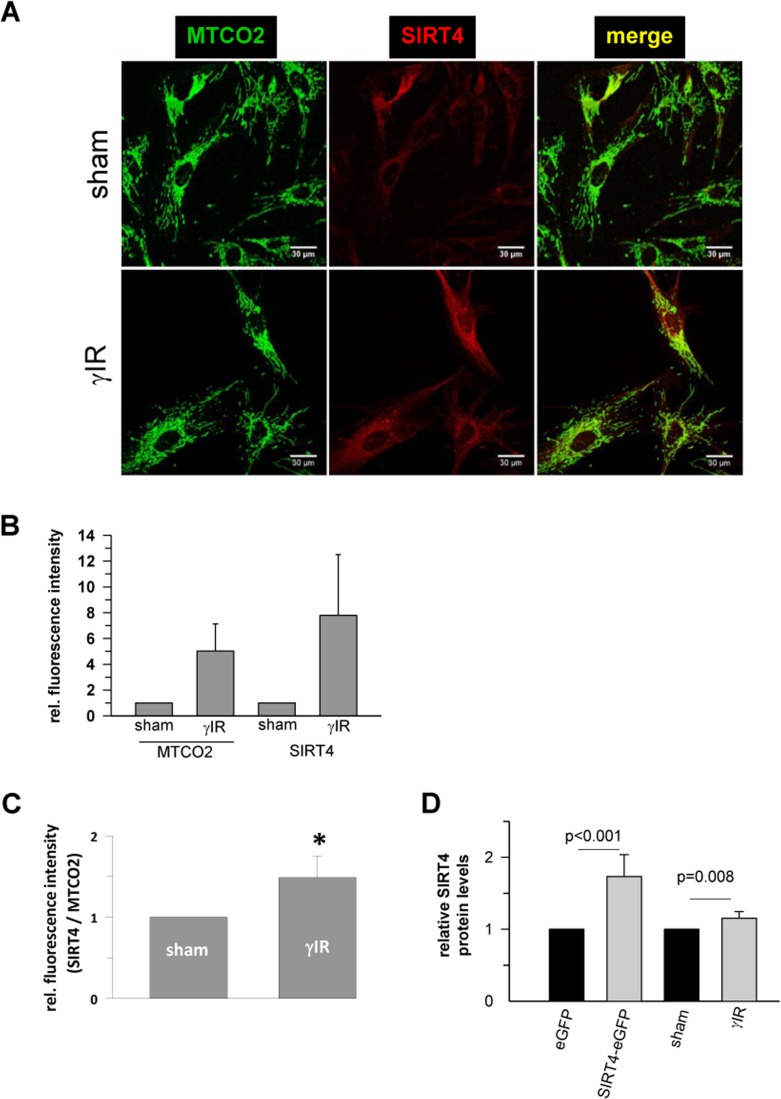
Confocal microscopy based analysis of the expression and colocalization of SIRT4 with the mitochondrial marker MTCO2 (mitochondrially encoded cytochrome C oxidase II) in γ-irradiated human dermal fibroblasts (**A**) Subcellular visualization of MTCO2 (green) and SIRT4 (red) in γ-irradiated (20 Gy) fibroblasts as compared to control (sham treated) cells. (**B**) Relative quantification of total MTCO2 and SIRT4 signal intensities in γ-irradiated cells as compared to control (sham treated) cells using the ImageJ software. (**C**) Total SIRT4 signal intensities were normalized to MTCO2 signals in γ-irradiated cells as compared to control (sham treated) cells. Mean ± s.d. from four independent experiments. Thirty to fifty cells were analysed per experiment and condition. To evaluate statistical significance, Mann-Whitney rank sum test was performed (*p<0.05). (**D**) ELISA-based quantification of relative SIRT4 protein levels (suppl. Material & Methods) in γ-irradiated fibroblasts as compared to sham treated cells. As comparison, relative SIRT4 levels were determined in HEK293 cells stably expressing SIRT4-eGFP *vs.* eGFP expressing control cells. To evaluate statistical significance, Mann-Whitney rank sum test was performed (n=5 independent experiments, mean ± s.d).

To test whether senescence-associated SIRT4 upregulation was cell-type specific, we next employed additional cellular models of premature senescence. We combined this analysis with gene array-based expression screens, which included all SIRT isoforms (suppl. Fig. 1), followed up by reassessment of the expression of mtSIRTs using quantitative real-time PCR (qRT-PCR) (Fig. 2). Interestingly, among the seven sirtuin family members, and in particular among the mtSIRTs SIRT3, SIRT4, and SIRT5, only SIRT4 was consistently and significantly upregulated in epithelial breast adenocarcinoma MCF7 cells undergoing stress-induced senescence. This result was observed regardless of whether senescence was triggered by mitotic spindle stress through depletion of the centrosomal protein TACC3 [22] or by treatment with gamma-irradiation (γIR) [40] (suppl. Fig. 1 and Fig. 2A,B). Additionally, we also subjected primary human dermal fibroblasts to γIR to trigger stress-induced senescence [41]. As indicated in Fig. 3 and suppl. Fig. 2 (as well as in Fig. 6A at the transcript level), γIR resulted in an increased protein levels of SIRT4, which colocalized with the mitochondrial marker MTCO2 (mitochondrially encoded cytochrome C oxidase II) as observed by confocal laser scanning microscopy. The relative fluorescence intensity of cellular SIRT4 signals (as normalized to MTCO2 signals) was significantly higher in γ-irradiated cells as compared to sham treated controls (Fig. 3C). ELISA-based quantification of total cell lysates revealed SIRT4 protein levels which were increased by approximately 20% in irradiated *vs.* non-irradiated cells (Fig. 3D). These findings show that cellular senescence, either of replicative or stress-induced nature, is accompanied in a cell type-independent manner by an increased SIRT4 expression in mitochondria.

### Downregulation of miR-15b is inversely associated with increased SIRT4 expression in cellular senescence

Currently, little is known about the regulation of SIRT4 expression. A major feature of senescent cells is altered gene expression that leads to growth arrest, apoptosis resistance, and induction of the SASP [8, 42], which is controlled by the mammalian target of rapamycin complex 1 (mTORC1) pathway [43]. Surprisingly, although mTORC1 activity is thought to promote cellular senescence and accelerated aging [44], SIRT4 expression is transcriptionally repressed by mTORC1 [45]. Given our contrary findings of a senescence-associated upregulation of SIRT4 (Fig. 1-3 and 6A), we hypothesized that small non-coding silencing RNAs (miRNAs) would function as inhibitors of SIRT4 expression. In accordance with this hypothesis, a role of miRNAs has been proposed in various senescence and aging model systems [46-51]. MicroRNAs represent potent regulators of gene expression networks by either degrading or translationally repressing one or more target mRNAs. This process enables cells to coordinate and fine-tune a variety of cellular processes or to modulate antagonistic phenotypes like malignant proliferation *vs.* senescence/cellular aging. As an example, increased *vs.* decreased levels of cell cycle regulating miRNAs from the miR-17-92 cluster are associated with malignant proliferation or oncogene-induced senescence, respectively [50, 52].

In order to assess the potential role of miRNAs in senescence-associated SIRT4 upregulation, we next performed a global, microarray-based comparison of corresponding miRNA- and mRNA expression profiles (both linked in suppl. Table 2) followed by relative miRNA quantification using qRT-PCR in our model systems. Our miRNA findings are based on an initial screen performed in MCF7 cells undergoing premature senescence upon TACC3 depletion (suppl. Table 1). We identified and verified a differential expression of various miRNAs, including members of the miR-17-92 and miR-106a-363 clusters, which became down-regulated upon cellular senescence (suppl. Table 1 and suppl. Fig. 3), consistent with the work of Hackl et al. [48]. Upregulated senescence-associated miRNAs included miR-146, which is linked to dampening of the SASP [46], and miR-125b, which is involved in the regulation of keratinocyte proliferation and differentiation [53]. In contrast, miR-22, a miRNA upregulated in human senescent fibroblasts targeting SIRT1 expression [54], was not identified as differentially expressed upon TACC3 depletion (suppl. Table 1), a finding consistent with insignificantly changed SIRT1 mRNA levels (suppl. Fig. 1).

**Figure 4:**
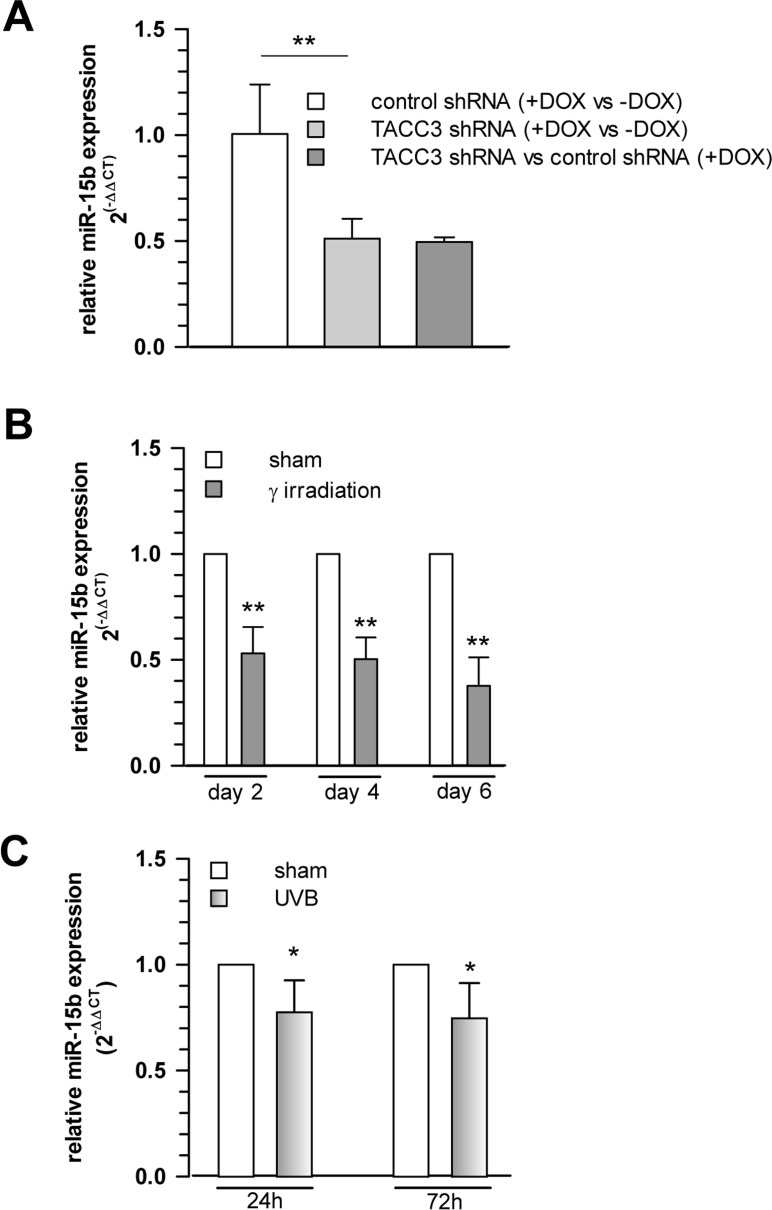
Cellular levels of miR-15b are decreased concomitant with upregulated SIRT4 gene expression in various aging models (**A**) Decreased miR-15b mRNA levels in MCF7 cells following induction of senescence through TACC3 depletion (analysis on day 4 of doxycycline-induced TACC3 shRNA expression; mean ± s.d. from three independent experiments). To evaluate statistical significance, ANOVA SNK were performed (**p<0.01). (**B**) Analysis of miR-15b levels in MCF7 cells following ionizing radiation (application of 20 Gy at day 0; mean ± s.d. from three independent experiments). (**C**) Primary human dermal fibroblasts (n=4 donors) were subjected to ultraviolet irradiation (100 J/m^2^ UVB per day) for a total of five days. Cells were harvested 24 and 72 h following the last irradiation and miR-15b levels were determined by qRT-PCR (mean ± s.d.). To evaluate statistical significance in (b) and (c), t-tests (n=3; **p<0.01) and Mann-Whitney rank sum tests (n=4; *p<0.05) were performed, respectively.

Interestingly, among the miRNAs analysed, we observed a robust and significant downregulation of miR-15b in TACC3 depleted or γ-irradiated senescent MCF7 cells (Fig. 4A,B), concomitant with a parallel upregulation of SIRT4 expression (Fig. 2), which represents a *bona fide* candidate target of miR-15b (suppl. Table 2 and suppl. Fig. 4). Similarly, and again in accordance with the SIRT4 expression status (Fig. 1B), miR-15b levels were also decreased in UV-irradiated or gamma-irradiated senescent primary human dermal fibroblasts (Fig. 4C and 6B). Lastly, consistent with our findings, up to fourfold decreased miR-15b levels were observed in various models of organismal aging as well as replicative cellular senescence [48, 49, 55, 56], the latter being in accordance with an increased SIRT4 expression in human fibroblasts undergoing replicative senescence (Fig. 1A).

### Inverse expression of miR-15b and SIRT4 *in-vivo* in photoaged human skin

**Figure 5:**
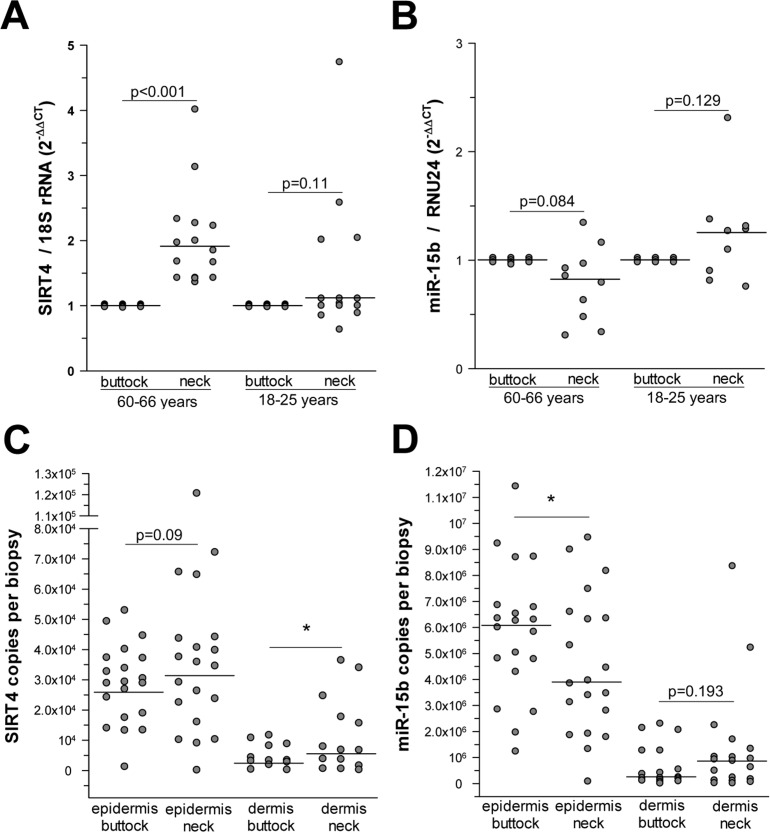
Increased SIRT4 expression is inversely associated with decreased miR-15b levels in human photoaged skin (**A**) SIRT4 expression analysis in human skin samples obtained from neck *vs.* buttock skin of the same individuals (age ranges: 18-25 years and 60-66 years; n=15-16 per group). (**B)** miR-15b expression analysis in human skin samples obtained from neck *vs*. buttock skin of the same individuals (age ranges: 18-25 years and 60-66 years; n=9-10 per group). (**C, D**) Determination of SIRT4 mRNA (**c**) and miR-15b (**d**) copy numbers in the epidermal (SIRT4: n=20; miR-15b: n=20) and dermal (SIRT4: n=12-14; miR-15b: n=17-19) compartments isolated from human photoaged skin (neck *vs.* buttock) of the same individuals (age range: 60-66 years). Median ± SEM; *p<0.05. To evaluate statistical significance, Wilcoxon signed rank tests were employed.

After establishing an inverse correlation between miR-15b and SIRT4 expression in various *in vitro* models of cellular senescence, we next asked whether an analogous scenario exists in organ aging. To answer this question we focused on photoaged human skin, in which the aging process is driven by UV radiation exposure (*i.e.,* photoaging) through mechanisms involving mitochondrial dysfunction [3], and which can be easily distinguished from intrinsically (chronological) aged skin by means of the SCINEXA^TM^ score [57] (suppl. Fig. 5 & suppl. Material and Methods). Interestingly, a highly significant increase of SIRT4 transcripts was detected in human skin biopsies obtained from photoaged skin (fold increase: 1.91 ± 1.23; median ± SEM, p<0.001; n=16) compared with non-photoaged skin of the same individuals aged 60-66 years or to skin of young individuals (n=15; age range 18-25 years) (Fig. 5A). In a similar trend, the same biopsies showed a decreased expression of miR-15b in photoaged skin samples of the older individuals (0.82 ± 0.11; median ± SEM) as compared with their intrinsically aged skin counterparts (Fig. 5B). This difference was significant when SIRT4 and miR-15b copy numbers were measured in a skin compartment specific manner. In these experiments, skin biopsies from twenty individuals (age range: 60-66 years) were subjected to dispase II treatment to enzymatically separate the epidermal and dermal compartments followed by a quantitative determination of the copy numbers of SIRT4 and miR-15b using internal standards (see Material & Methods). We found that SIRT4 levels were significantly increased in the dermis of photoaged human skin as compared with non-photoaged skin (6.75×10^3^ ± 3.31×10^3^ *vs*. 3.37×10^3^ ± 1.14×10^3^; median ± SEM; *p<0.05) (Fig. 5C). In addition, there was a trend towards higher SIRT4 copy numbers in photoaged skin, if epidermal compartments were compared with each other. Analysis of miR-15b expression revealed that it was highest in the epidermis as compared with the dermis, thus confirming a previous report [58]. Importantly, comparison of photoaged *vs*. non-photoaged skin revealed the following pattern: In the photoaged epidermis significantly less miR-15b copy numbers were detected as compared with non-photoaged epidermis (3.91×10^6^ ± 0.60×10^6^ *vs*. 6.13×10^6^ ± 0.57×10^6^; median ± SEM; *p<0.05), and for the two dermal compartments a trend towards decreased copy numbers in photoaged skin was evident (Fig. 5D). Based on these results we conclude that miR-15b downregulation in the epidermis may contribute to aging-associated SIRT4 expression. Given the high levels of miR-15b in the epidermis (Fig. 5D) [58], it is tempting to speculate that the epidermis and dermis communicate with each other *via* intercellular transport of miRNAs. Such cell-cell communication could be mediated by keratinocyte microvesicles (containing besides proteins and nucleic acids also miRNAs), which have been recently shown to affect gene and protein expression in fibroblasts [59].

Alternatively, photoaging-associated SIRT4 increase in the dermis may occur independently from miR-15b expression, possibly associated with downregulation of other miR-15/16 family members as observed in gamma-irradiated human dermal fibroblasts (suppl. Fig. 6). Nevertheless, in all *in vitro* models studied an inverse correlation between SIRT4 and miR-15b was observed to occur during cellular senescence and, irrespective of the precise link between SIRT4 and miR-15b (cell autonomous or paracrine regulation), also in photoaging of human skin and thus in organ aging. Of note, Dong et al. observed inverse alterations of transcript levels of mtSIRTs, i.e. SIRT4 (increased) *vs*. SIRT3 (decreased) in normal human epidermal keratinocytes upon UVB exposure [60]. In addition, expression of SIRT3 (besides that of SIRT1 and SIRT7) seems to be decreased in photoaged/UV-irradiated skin as compared to non-irradiated control samples [61, 62].

### MicroRNA-15a is also stress responsive and downregulated uponγirradiation in primary human dermal fibroblasts

miR-15b was the only regulated and senescence-linked miRNA in our miRNA screen (suppl. Table 1) with a uniquely conserved binding site within the 3′-untranslated region (UTR) of the *SIRT4* gene in vertebrates (suppl. Fig. 4). However, it should be noted that this binding site can also be targeted by additional miRNAs (miR-15a/16/195/424/497; suppl. Fig. 4) which recognize the same seed sequence [63]. In addition to miR-15b human dermal fibroblasts also express miR-15a and miR-16-1 at high and comparable copy numbers, whereas the expression levels of miR-16-2, miR-195, and miR-497 were clearly lower (suppl. Fig. 6). In this regard, miR-195 levels are elevated rather than downregulated upon replicative senescence [64]. Interestingly, besides miR-15b, miR-15a (but not miR-16-1) was also stress-responsive and significantly downregulated upon γIR treatment (suppl. Fig. 6). Thus, SIRT4 expression may be repressed by both miR-15b and miR-15a during cellular senescence and aging, probably in a cell-type and senescence stimulus dependent manner.

Members of the miR-15/16 group are genomically embedded in two host genes, SMC4 (structural maintenance of chromosomes 4) and pseudogene DLEU2 (deleted in leukemia 2). To gain insight into the regulation of miR-15/16 family members during photoaging, we determined the transcript levels of SMC4 and DLEU2, which can be assumed to be coexpressed with miR-15b/16-2 and miR-15a/16-1, respectively [63]. However, as depicted in suppl. Fig. 7, and in contrast to decreased miR-15b levels (Fig. 5B,D), the expression of both SMC4 and DLEU2 was increased in photoaged skin (n=13; age range 60-66 years) as compared to non-photoaged skin of the same individuals. Thus, the expression of miR-15/16 family members is not co-regulated with their host genes during aging. Of note, these microRNAs are subject to global cell stress and p53 regulation [63], and increased miR-15b expression was shown to correlate with tumor cell proliferation and apoptosis [65]. In this regard, miR-15 and miR-16 are direct transcriptional targets of E2F1 and become induced during mitogenic signaling to prevent replicative stress [66, 67]. Expression of E2F1 is downregulated during cellular senescence [68], and a 3.5- to 5.1-fold downregulation of E2F1 transcipts was observed by microarray analysis in spindle stress-induced and γIR-triggered senescence, respectively (data not shown). Concomitantly, E2F1 protein levels were strongly decreased (suppl. Fig. 8). It is thus tempting to speculate that E2F1 is a major regulator of the miR-15/16 – SIRT4 axis during cellular senescence, with decreased E2F1 causing reduced miR-15/16 levels resulting in increased SIRT4 expression. Interestingly, miR-17-92 represents another major miRNA cluster that is consistently downregulated in senescence and human aging [48] (suppl. Fig. 3). Given that E2F1 induces expression of miR-17-92 [69], it might be that downregulation of the miR-17-92 cluster, similar to miR-15/16, is based on decreased E2F1 levels as well.

### MicroRNA-15b directly inhibits stress-induced expression of SIRT4

**Figure 6:**
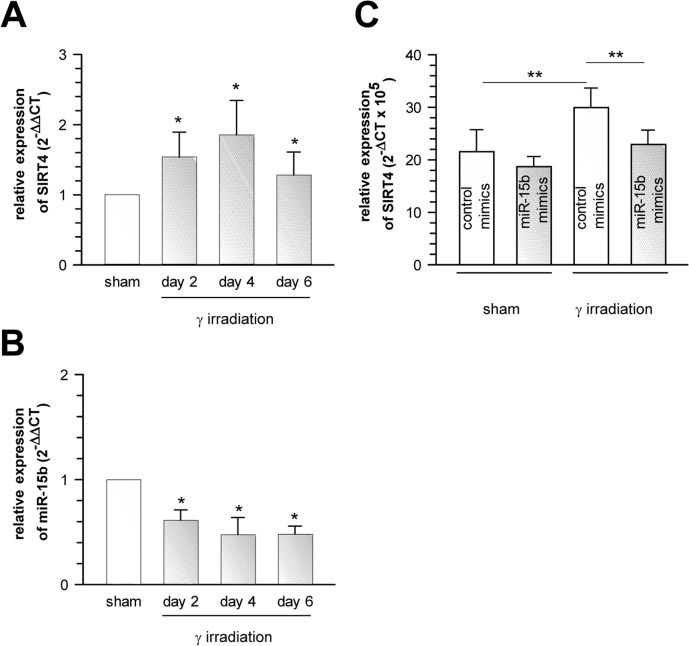
Suppression of senescence-associated SIRT4 upregulation in γ-irradiated primary human dermal fibroblasts by oligonucleotides mimicking the function of endogenous miR-15b Concomitant upregulation of SIRT4 expression (**A**) and downregulation of miR-15b levels (**B**) in human dermal fibroblasts two, four, and six days upon γ-irradiation (single dose of 20 Gy; n=6, mean ± s.d.). To evaluate statistical significance, ANOVA on ranks were performed (*p<0.05). (**C**) Extrinsically applied miR-15b mimics inhibit the senescence-associated upregulation of SIRT4 mRNA levels in human dermal fibroblasts upon γ-irradiation (single dose of 20 Gy) as compared to irradiated cells transfected with control mimics (n=5 independent experiments, mean ± s.d.). To evaluate statistical significance, ANOVA SNK were performed (**p<0.01).

To assess if miR-15b directly regulates SIRT4 mRNA levels, we exogenously applied oligonucleotides mimicking endogenous miR-15b function (miR-15b “mimics”) to the MCF7 model of spindle stress-triggered senescence as well as to gamma-irradiated senescent human dermal fibroblasts. In these experiments, miR-15b mimics dampened basal SIRT4 mRNA levels and inhibited senescence-associated upregulation of SIRT4 transcripts (suppl. Fig. 9A and Fig. 6C). Moreover, by employing luciferase assays we show that the 3′UTR of human SIRT4 is a direct target of miR-15b, as miR-15b mimics significantly inhibited the relative luciferase activity of a transfected SIRT4 – 3′-UTR construct, but not, when the SIRT4 – 3′-UTR was mutated within the seed sequence for miR-15b (suppl. Fig. 9B). These results show that miR-15b mimics are *per se* sufficient to directly target SIRT4 expression. However, we cannot exclude the possibility that additional miRNAs, like miR-15a, which underlie stress regulation (suppl. Fig. 6) and potentially recognize the 3′-UTR of the *SIRT4* gene, are also of functional relevance for the regulation of SIRT4 expression.

### Inhibition of miR-15b promotes mitochondrial ROS generation and reduces mitochondrial membrane potential in a SIRT4-dependent manner

**Figure 7:**
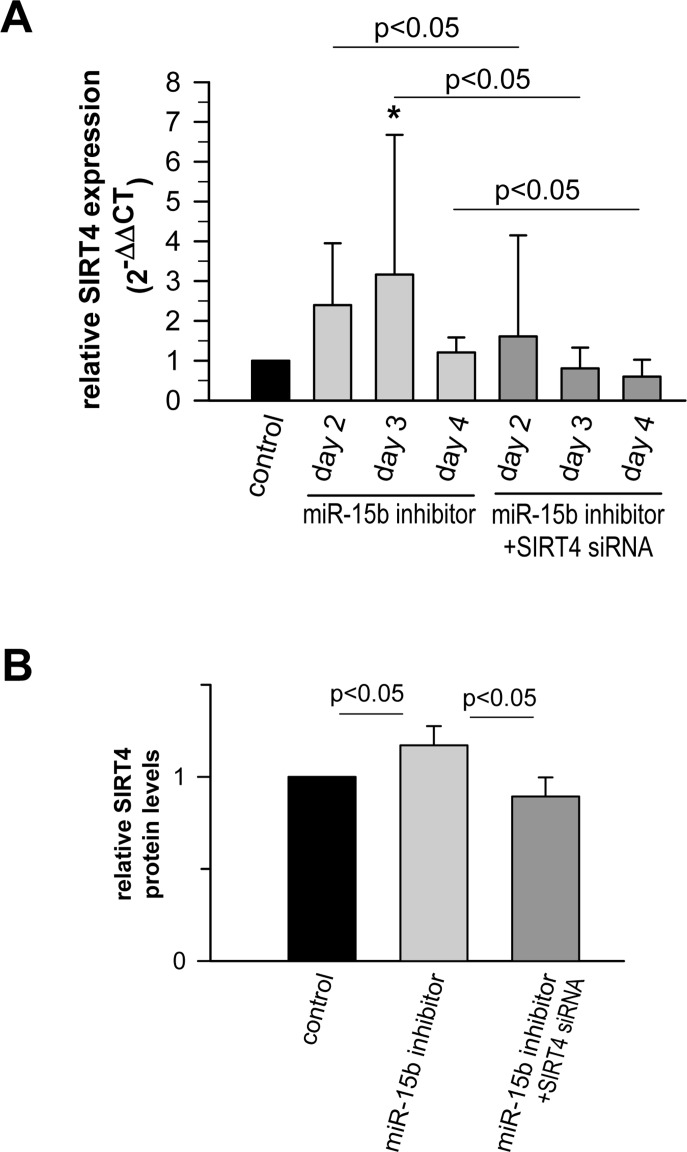

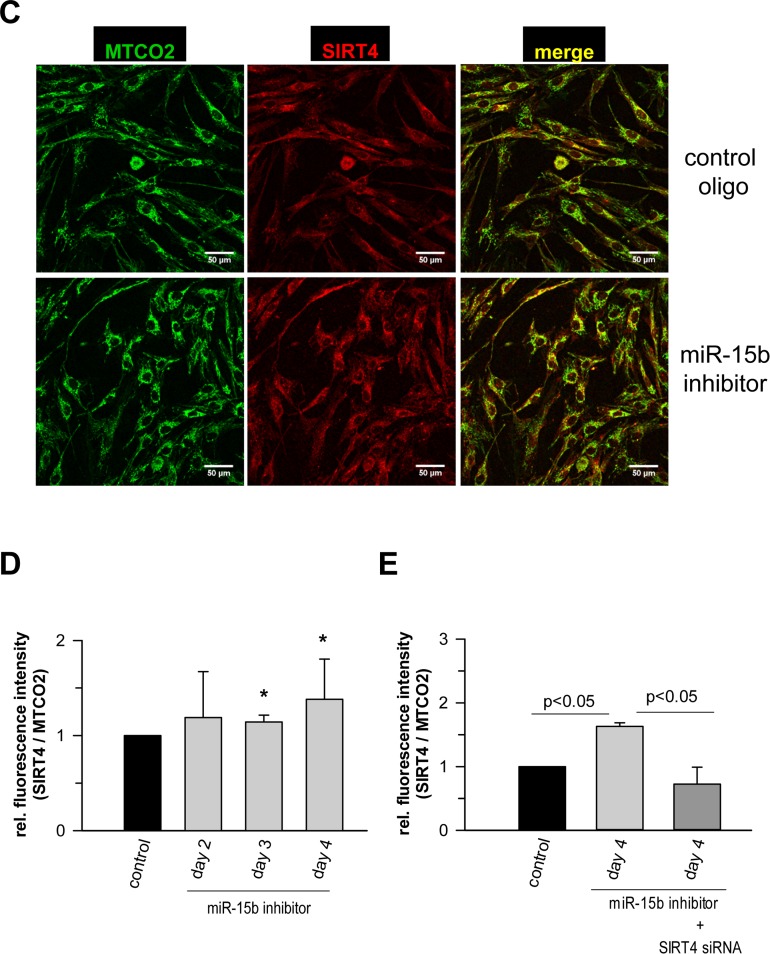
Analysis of the expression of SIRT4 and its colocalization with the mitochondrial marker MTCO2 in human dermal fibroblasts upon transfection of miR-15b inhibitors (**A**) Modulation of SIRT4 expression *via* transfection of miR-15b inhibitors in the absence or presence of siRNA duplexes against SIRT4 (mean ± s.d. from nine to twelve experiments). (**B**) ELISA-based quantification of relative SIRT4 protein levels (suppl. Material & Methods) in fibroblasts transfected with miR-15b inhibitors in the absence or presence of siRNA duplexes against SIRT4 (mean ± s.d. from four experiments). To evaluate statistical significance, ANOVA SNK was performed. (**C**) Confocal microscopy-based visualization of the mitochondrial marker MTCO2 (green) and SIRT4 (red) in miR-15b inhibitor-transfected fibroblasts as compared to control cells. Total SIRT4 signal intensities were normalized to MTCO2 in miR-15b inhibitor-transfected cells in the absence (**D**) or presence (**E**) of siRNA duplexes against SIRT4 using ImageJ software. Mean ± s.d. from four (**D**) and three (**E**) independent experiments. Thirty to fifty cells were analysed per experiment and condition. To evaluate statistical significance, ANOVA on ranks Dunn´s tests were performed (*p<0.05).

**Figure 8:**
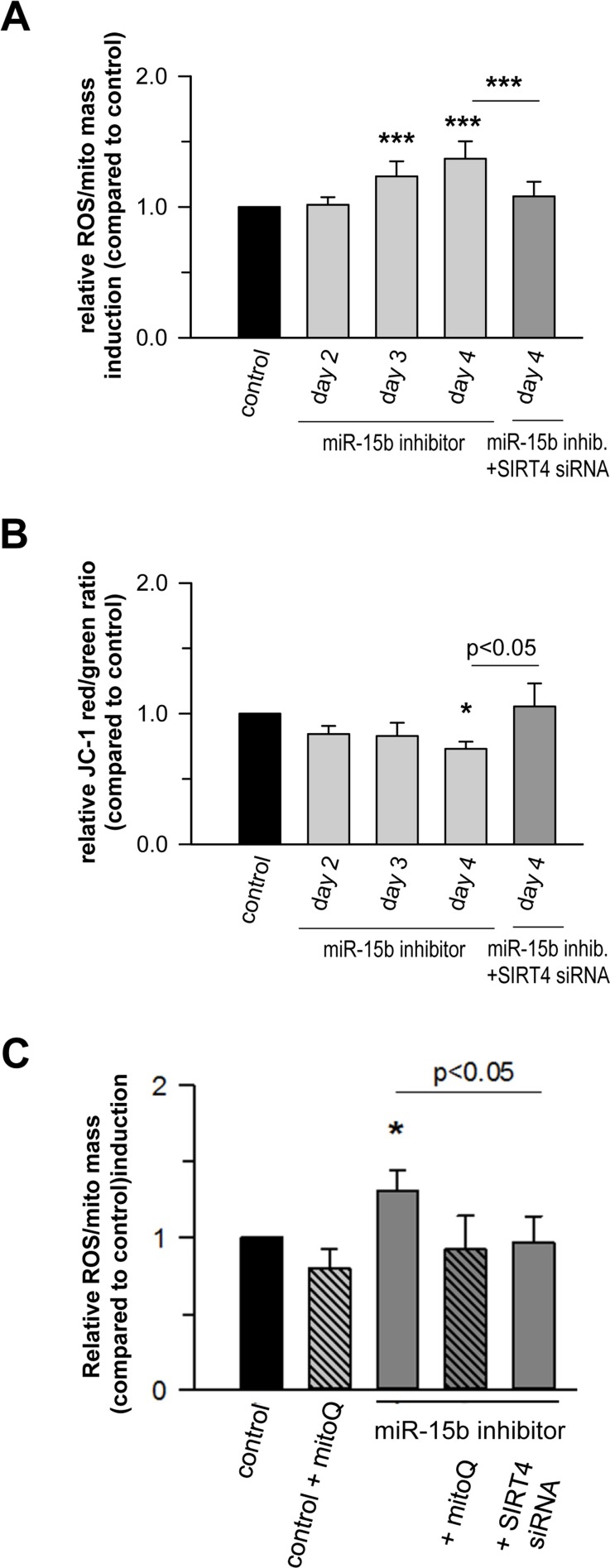
Mir-15b regulates mitochondrial ROS generation and the mitochondrial membrane potential in a SIRT4-dependent manner in primary human dermal fibroblasts Mitochondrial ROS levels (**A**) and the mitochondrial membrane potential (**B**) were determined by staining with cell-permeable dyes and flow cytometric analysis as described in Materials & Methods and by Kalfalah *et al.* [102]. Shown are mean ± s.d. values from four to six experiments. (**C**) Mitochondrial ROS levels were determined in the presence of the mitochondria-specific antioxidant mitoQ [72]. Cells were analyzed four days following transfection of miR-15b inhibitors in the absence or presence of siRNA duplexes against SIRT4. Depicted are mean ± s.d. values from four to six experiments. To evaluate statistical significance, ANOVA on ranks Dunn´s tests were performed (*p<0.05; ***p<0.001).

Recent studies showed that miR-15b can act as a negative modulator of cellular ATP levels and mitochondrial function by targeting ADP-ribosylation factor-like 2 (Arl2) [70]. SIRT4 and Arl2 are not the only miRNA targets transcriptionally regulated during cellular senescence and implicated in the regulation of mitochondrial energy metabolism. Using the aging mouse brain model, Li et al. observed an upregulation of a panel of miRNAs predicted to target key genes involved in oxidative phosphorylation, including the mitochondrial complex IV and F0F1 ATPase [71]. Given these findings we next assessed the role of the miR-15b – SIRT4 axis in mitochondrial function of primary human dermal fibroblasts. Transfection of fibroblasts with miR-15b inhibitors, which caused a significant upregulation of SIRT4 at the transcript (Fig. 7A), protein (Fig. 7B) and protein/mitochondrial localization level (Fig. 7C-E), resulted in a concomitant increase in mitochondrial ROS levels and a decrease of the mitochondrial membrane potential (Fig. 8A, B). Importantly, co-transfection of miR-15b inhibitors with siRNA duplexes against SIRT4 (Fig. 8A, B) or co-treatment with the mitochondria-specific antioxidant mitoQ [72] (in the case of mtROS levels; Fig. 8C) prevented these changes, indicating that miR-15b negatively impacts on mitochondrial function in a SIRT4-dependent manner.

**Figure 9:**
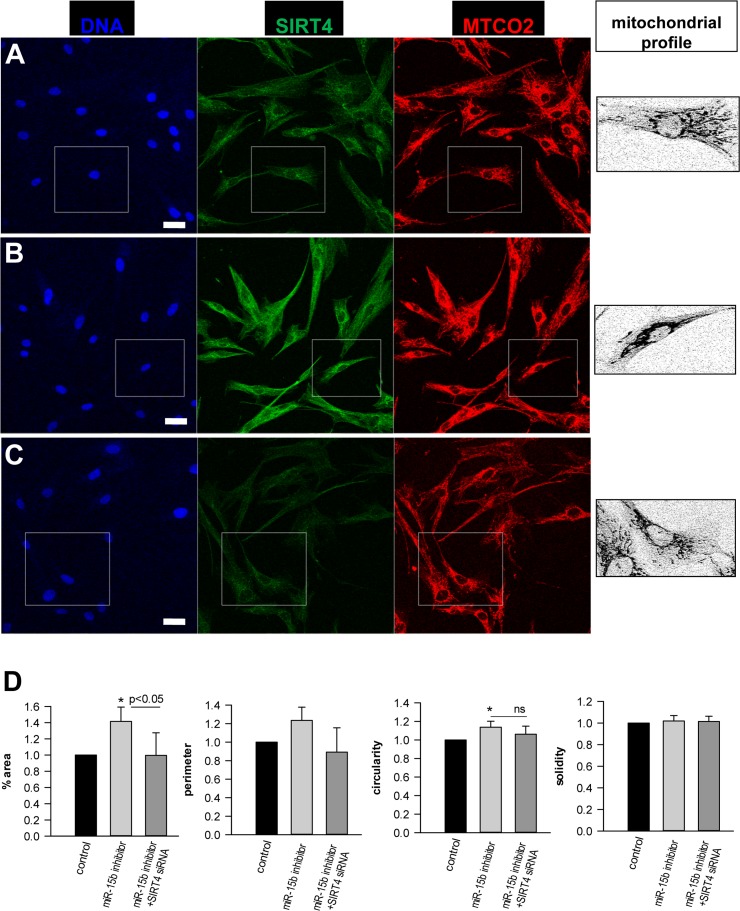
Analysis of the effect of the miR-15b – SIRT4 axis on mitochondrial morphology and fragmentation Human dermal fibroblasts were transfected with control oligos (**A**) or miR-15b inhibitor oligos in the absence (**B**) or (**C**) presence of siRNA duplexes against SIRT4. Four days later, cells were stained with SIRT4 and MTCO2 specific antibodies. Confocal laser scanning microscopy and an ImageJ macro were employed to analyse fold changes in mitochondrial morphology-related parameters, including %area (total mitochondrial area/total cell area), mitochondrial perimeter, circularity, and solidity. Approximately two hundred cells were analysed per experimental condition. To evaluate statistical significance, ANOVA SNK were performed (*p<0.05). Bar: 20 μm.

At a molecular level, SIRT4 may modulate mitochondrial oxidative capacity and function *via* ADP-ribosylation and subsequent inhibition of its target glutamate dehydrogenase (GDH), thereby linking amino acid metabolism with oxidative phosphorylation [35]. A direct involvement of SIRT4 in ROS generation is likely given the finding that the SIRT4-GDH axis modulates mitochondrial permeability *via* the mitochondrial permeability transition pore (mPTP) [73], which directly influences the mitochondrial membrane potential [74]. However, GDH activity was not altered in fibroblasts upon transfection with miR-15b inhibitors (suppl. Fig. 10). Alternatively, the miR-15b – SIRT4 axis might affect ROS generation and mitochondrial dysfunction (Fig. 8) *via* another metabolic gate keeper, the pyruvate dehydrogenase (PDH) complex, because SIRT4 was recently found to inhibit PDH *via* a lipoamidase activity [37]. This assumption is consistent with the observation that defects in the PDH complex are associated with increased ROS (superoxide) accumulation and age-related disorders [75]. Indeed, preliminary data indicate that inhibition of miR-15b impedes PDH activity in human dermal fibroblasts in a SIRT4-dependent manner (Lang et *al.*, unpublished). Given that ROS affects mitochondrial fragmentation [76, 77] we next addressed the impact of the miR-15b – SIRT4 axis on mitochondrial morphology. Employing confocal imaging (SIRT4/MTCO2) and ImageJ-based quantification, the fold changes in mitochondrial morphology-related parameters (%area, perimeter, circularity, and solidity) were analysed in dermal fibroblasts four days after one-time transfection with miR-15b inhibitors +/− siRNA duplexes against SIRT4. Interestingly, as indicated in Figure 9, inhibition of miR-15b led to increases in average mitochondrial area (in relation to the average cellular size), perimeter, and circularity which were ameliorated when SIRT4 was depleted. However, we did not observe significant changes in mitochondrial solidity, indicating that the mitochondrial dynamics in terms of connectivity and fragmentation was greatly unchanged. The latter could be supported by preliminary experiments using Mitotracker stainings (data not shown).

Collectively, this study and other studies indicate that SIRT3 and SIRT5 promote mitochondrial energy production, whereas SIRT4 has the opposite effect [34, 73, 78, 79]. SIRT3 and SIRT5 may thus antagonize metabolic abnormalities occurring in cellular senescence and organismal aging, whereas SIRT4 has opposite effects. Mechanistically, SIRT3 deacetylates and thereby activates mitochondrial HMG-CoA synthetase 2 required for ketone body production [80] and functions as a metabolic tumor suppressor [81]. Moreover, SIRT3 deficiency causes a severe dysregulation of mitochondrial protein acetylation which then contributes to the development of the metabolic syndrome [82]. SIRT3 and its mitochondrial acetyl-proteome may therefore be considered as an important network in metabolic homeostasis [83]. SIRT5 has been described to positively regulate the urea cycle [84] and to control glycolysis *via* lysine malonylation of glyceraldehyde 3-phosphate dehydrogenase [85]. In contrast, SIRT4, which is induced by DNA damaging agents and exhibits tumor-suppressive activity [86, 87], negatively impacts on gene expression and various metabolic processes in mitochondria. In addition to GDH and PDH, SIRT4 also targets malonyl CoA decarboxylase and thereby regulates lipid homeostasis at the protein [36] and transcript level [79]. Accordingly, knock down of SIRT4 *in vivo* leads to an improved fatty acid oxidation and mitochondrial function [88].

### The miR-15b - SIRT4 axis may regulate expression of nuclear encoded mitochondrial genes

**Figure 10:**
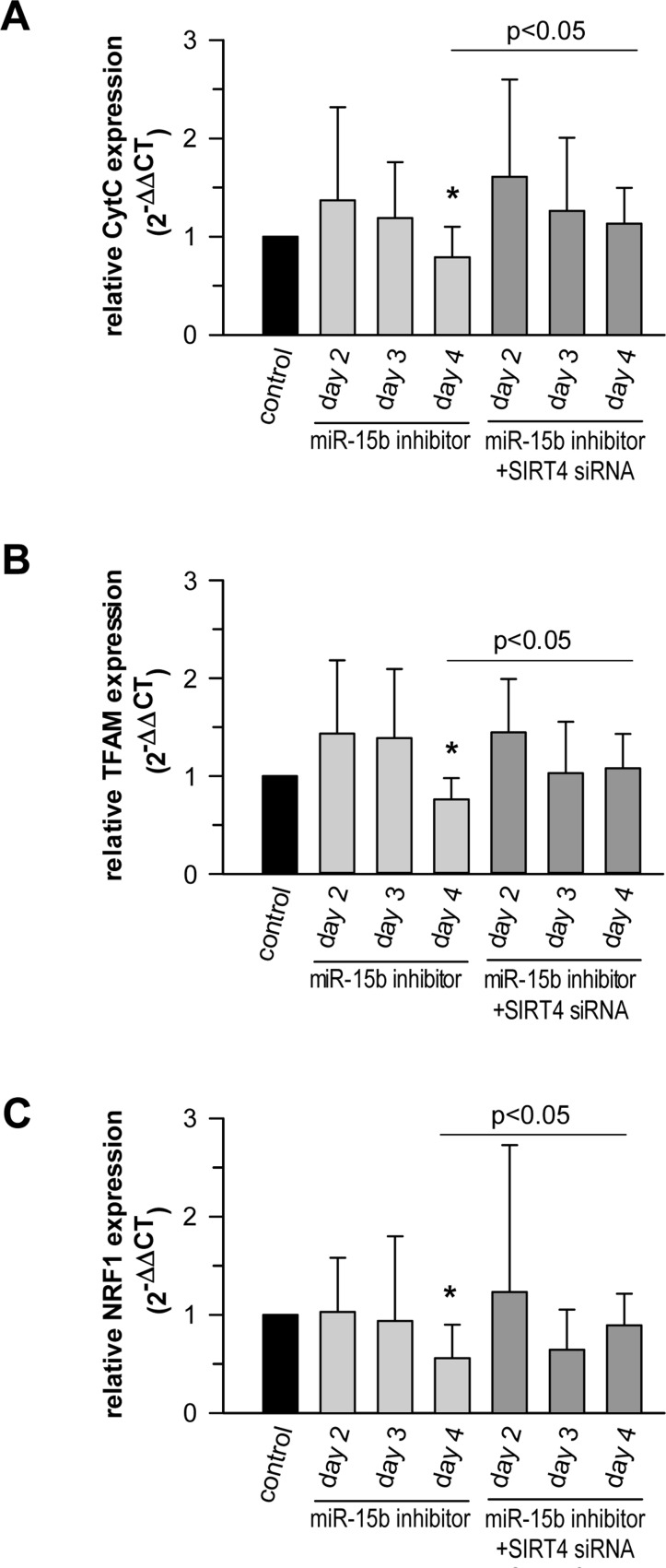
The miR-15b - SIRT4 axis regulates the expression of nuclear encoded mitochondrial genes Inhibitors against miR-15b were transfected in primary human dermal fibroblasts in the absence or presence of siRNA duplexes against SIRT4. Transcript levels of CytC (**A**), TFAM (**B**), and NRF1 (**C**) were determined by qRT-PCR. Shown are mean ± s.d. values from nine to thirteen experiments. To evaluate statistical significance, ANOVA on ranks Dunn´s tests were performed (*p<0.05).

It has been reported that overexpression of SIRT4 elicits an AMPK-dependent “retrograde” signaling response from mitochondria to the nucleus thereby inhibiting expression of nuclear encoded key genes involved in mitochondrial biogenesis [79]. We therefore tested the impact of the miR-15b – SIRT4 axis on the expression of cytochrome C (CytC), a major component of the respiratory chain, as well as TFAM (TFAM, transcription factor A, mitochondrial) and NRF1 (NRF1, nuclear respiratory factor 1), which are both critically involved in mitochondrial gene expression [89, 90] and the antioxidant response [91]. Primary human dermal fibroblasts transfected with miR-15b inhibitors displayed a significant SIRT4 increase as early as three days after transfection (Fig. 7). Interestingly, under these conditions transcript levels of CytC, TFAM, and NRF1 were significantly reduced by approximately 21 to 44% by day 4 (Fig. 10A-C). The decrease in transcript levels could be prevented when miR-15b inhibitors were co-transfected with siRNA duplexes against SIRT4 showing that it is SIRT4 dependent (Fig. 10A-C). Thus, consistent with the findings of Ho et al. [79], increased SIRT4 expression may antagonize the expression of important nuclear encoded mitochondrial proteins. These results further support the concept of a causal relationship between the miR-15b – SIRT4 axis and ROS generation / mitochondrial dysfunction.

### Inhibition of miR-15b impacts on the expression of components of the SASP in a SIRT4-dependent and -independent manner

**Figure 11:**
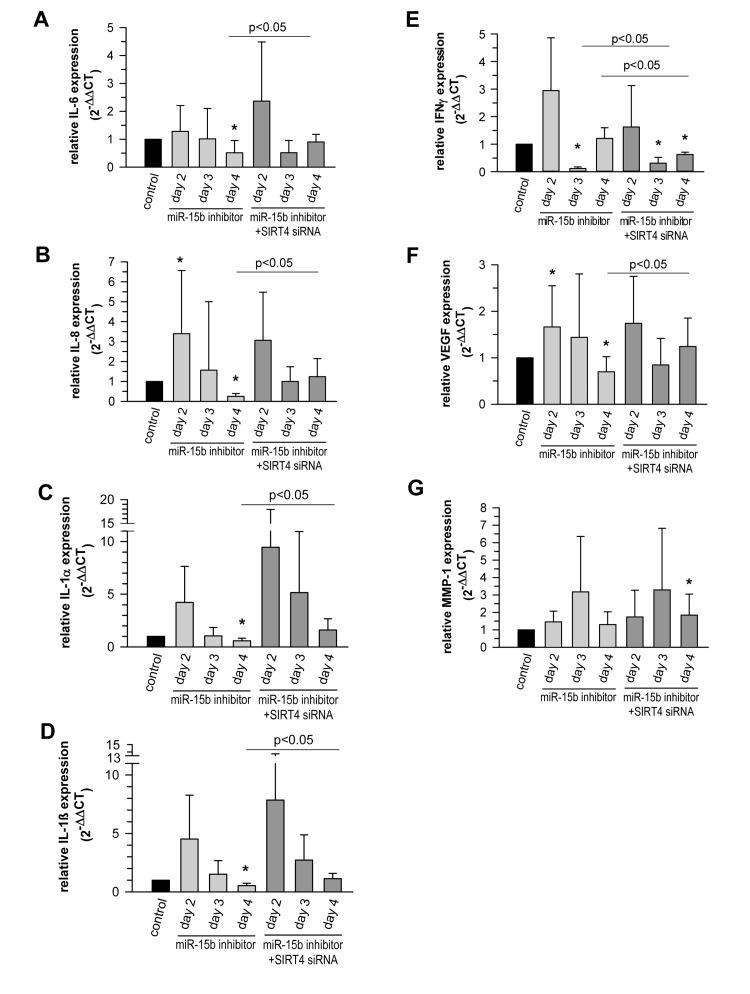
The miR-15b - SIRT4 axis regulates the expression of components of the senescence-associated secretory phenotype (SASP) Inhibitors against miR-15b were transfected in primary human dermal fibroblasts in the absence or presence of siRNA duplexes against SIRT4. Transcript levels of Interleukin-6 (IL-6) (**A**), Interleukin-8 (IL-8) (**B**), Interleukin-1α (IL1α) (**C**), Interleukin-1β (IL1β) (**D**), Interferon γ (IFNγ) (**E**), vascular endothelial growth factor (VEGF) (**F**), and matrix metalloproteinase-1 (MMP1) (**G**) were analysed by qRT-PCR. Shown are mean ± s.d. values from nine to thirteen experiments (except **C**, **D**, and **E**, where 5-6 independent experiments were performed). To evaluate statistical significance, ANOVA on ranks Dunn´s tests were performed (*p<0.05).

Senescent cells produce and secrete various autocrine- or paracrine-acting factors, including pro-inflammatory (e.g. IL-6) and pro-migratory (e.g. IL-8) cytokines, growth factors (e.g. VEGF), and proteases like matrix metalloproteinase-1 (MMP-1), collectively known as the SASP [14, 15, 17, 92-94]. MicroRNAs play an important role in SASP regulation and e.g. prevent an overproduction of inflammatory cytokines and therefore persistent inflammatory responses [17]. This is e.g. illustrated by the senescence associated up-regulation of miR-146b (suppl. Table 1) which serves to tune down the expression of Interleukin-6 (IL-6) and Interleukin-8 (IL-8) *via* downregulating IRAK1 expression and thus NFκB activity [46]. We therefore tested whether the miR-15b – SIRT4 axis is of functional relevance for the expression of well-known components of the SASP in primary human dermal fibroblasts. Interestingly, miR-15b inhibitors displayed a biphasic impact on the expression of VEGF and the cytokines analysed. We observed an increased expression at day 2 upon transfection of miR-15b inhibitors followed by a clear decrease of transcript levels (31-80%) at day 3 (IFNγ) and day 4 (IL-1α, IL-1β, IL-6, IL-8, and VEGF) (Fig. 11A-F). The latter decrease was SIRT4 dependent as the expression of all factors was ameliorated when miR-15b inhibitors were co-transfected with siRNA duplexes against SIRT4 (Fig. 11A-F). Of note, the initial rise in cytokine transcript levels upon miR-15b inhibition could be potentially due to increased IRAK2-NFκB signaling, given that IRAK2 represents a predicted target gene of miR-15b [95] (A. Lang and R. Piekorz, unpublished). Taken together, miR-15b may regulate the SASP, which is typically NFκB driven [96], at two levels, negatively *via* IRAK2, comparable with miR146-induced dampening of IL-6 and IL-8 expression *via* IRAK1 targeting [46], and positively *via* SIRT4 downregulation.

The expression of MMP-1, a SASP component that is typically upregulated in photoaged human skin *in vivo* [3], was also increased up to 3.2-fold in fibroblasts upon transfection of miR-15b inhibitors (Fig. 11G). However, this effect was SIRT4-independent, since co-transfection of miR-15b inhibitors and SIRT4-specific siRNA duplexes failed to alter the expression kinetics of MMP-1. This observation is of particular interest, because increased MMP-1 expression in photoaged skin is believed to cause profound degradation of collagen fibers and the subsequent development of coarse wrinkles, i.e. a clinical hallmark of photoaged skin [1, 2]. Given our observation that decreased miR-15b levels are also present in photoaged human skin *in vivo,* we thus propose a model for skin aging in which reduced miR-15b disturbs mitochondrial function and contributes to the development of a SASP including an increased production of MMP-1. Further studies employing conditional mouse models deficient in SIRT4 expression and/or function in the skin are necessary to test this hypothesis and to define more closely SIRT4-dependent and -independent pathways downstream of miR-15b.

Taken together, this work identifies miR-15b as a novel regulator of stress induced increase of SIRT4 expression and thereby links the miR-15b – SIRT4 axis to senescence associated mitochondrial (dys)function/redox homeostasis and SASP regulation.

## MATERIAL AND METHODS

### Ionizing γ-irradiation (γIR) of primary human dermal fibroblasts

Primary human dermal fibroblasts isolated from the foreskin of two different donors (F623 and F357) were cultured on 10 cm plates as described below. Cells were trypsinized and exposed to γIR (20 Gy) using a Gammacell 1000 Elite irradiator (Nordion International, Inc., Fleurus, Belgium). After irradiation, cells were plated at a density of 3.6×10^3^/cm^2^ and 1.8×10^3^/cm^2^ (controls, day 2 and days 4 and 6, respectively) and 7.3×10^3^/cm^2^ (20 Gy) and harvested for mRNA analysis on days 2, 4, and 6 after treatment.

### RNA extraction from cultured cells and cells subjected to UV or ionizing γ-irradiation and concomitant quantitative real-time PCR analysis (mRNA and miRNA)

Cell lysates were homogenized using Qiashredder spin columns (Qiagen, Hilden, Germany). Total RNA without small RNAs (> 200 bp) was extracted using the RNeasy Plus Mini Kit (Qiagen, Hilden, Germany) following the standard manufacturers protocol. Total RNA containing small RNAs was isolated using the RNeasy Plus Mini Kit (Qiagen, Hilden, Germany) following the supplementary protocol “Purification of total RNA containing miRNA from animal cells using the RNeasy Plus Mini Kit”. Nucleic acid concentrations were measured at 260 nm by spectrophotometry using a NanoDrop 1000 Spectrophotometer (Thermo Scientific, Wilmington, DE, USA). Extracted RNA samples were stored at −80 C.

For relative quantification of mRNAs, total RNA was reverse transcribed to cDNA using the ImProm-II™ Reverse Transcription System (Promega, Madison, WI, USA) according to the manufacturer's instructions. In brief, up to 1 μg RNA was reverse transcribed to cDNA in a final volume of 20 μl using Oligo (dT)_15_ primers (0.5 μg/reaction) and 3.75 mM MgCl_2_. Each quantitative real-time PCR reaction mixture (20 μl) contained 1 μl of RT product (cDNA transcribed from 50 ng of total RNA), 10 μl of TaqMan® Universal PCR Master Mix (Applied Biosystems, Austin, TX, USA) and 1 μl of the appropriate TaqMan® Gene Expression Assay (Applied Biosystems, Austin, TX, USA) containing primers and probe for the mRNA of interest. The mixture was initially incubated at 95°C for 10 min, followed by 40 cycles of 95°C for 15 s and 60°C for 60 s. PCR reactions were carried out on a 7500 Real-Time PCR System (Applied Biosystems, Austin, TX, USA) in triplicate. Samples were normalized relative to human large ribosomal protein (RPLPO) which served as endogenous control. mRNA levels were compared as 2^−▵Ct^ values or relative gene expression levels were calculated using the 2^−▵▵Ct^ method [97].

For relative quantification of miRNAs, total RNA was reverse transcribed using the TaqMan MicroRNA Reverse Transcription Kit (Applied Biosystems, Austin, TX, USA) according to the manufacturer's protocol. 10 ng of total RNA in a total volume of 15 μl were transcribed to cDNA using specific primers for each respective miRNA. The reverse transcription (RT) reaction was performed by sequential incubation at 16 C for 30 min, 42 C for 30 min, and 85 C for 5 min. Each quantitative real-time PCR reaction mixture (20 μl) contained 1.33 μl of RT product, 10 μl of TaqMan® Universal PCR Master Mix No AmpErase® UNG (Applied Biosystems, Austin, TX, USA) and 1 μl of the appropriate TaqMan® MicroRNA Assay (Applied Biosystems, Austin, TX, USA) containing primers and probe for the miRNA of interest. TaqMan® MicroRNA assays were used as follows. The mixture was initially incubated at 95 C for 10 min, followed by 40 cycles of 95 C for 15 s and 60 C for 60 s. PCR reactions were carried out on a 7500 Real-Time PCR System (Applied Biosystems, Austin, TX, USA) in triplicate. Samples were normalized relative to human small nucleolar RNA RNU38b or RNU24, which served as endogenous control. mRNA levels were compared as 2^−▵Ct^ values or relative gene expression levels were calculated using the 2^−▵ ▵Ct^ method [97].

TaqMan probes used were: SIRT4: Part No.: 4331182, assay-ID: Hs00202033_m1*; RPLPO: Part No.: 4331182, assay-ID: Hs99999902_m1*; hsa-mir-15b: Part No.: 4427975, assay-ID: 000390; RNU38B (for analysis of cultured cells): Part No.: 4427975, assay-ID: 001004; RNU24 (for analysis of total skin samples): Part No.: 4427975, assay-ID: 001001. Results are given as means ± s.d. P-values below 0.05 were considered significant.

### Analysis of gene expression of components of the SASP and nuclear encoded mitochondrial genes in primary human dermal fibroblasts

qRT-PCR analysis of matrix metalloproteinase-1 (MMP1) was performed essentially as described above by the TaqMan assay (part No.: 4331182, assay-ID: Hs00899658_m1*). Expression of further components of the SASP (IL-6, IL-8, vascular endothelial growth factor [VEGF], IL-1β, IL-1β, and IFNγ) as well as nuclear encoded mitochondrial genes (cytC, cytochrome C; TFAM, transcription factor A-mitochondrial; NRF1, nuclear respiratory factor 1) [79] was performed using the Power SYBR® Green PCR Master Mix (Applied Biosystems, Austin, TX, USA). PCR primer used were: IL-6 (P05231, 5′-AAAGAGGCACTGGCAGAAAA-3′ and 5′-CAGGGGTGGTTATTGCATCT-3′); IL-8 (P10145, 5′-TTTCCACCCCAAATTTATCA-3′ and 5′-AACCCTACAACAGACCCACA-3′); VEGF (P15692, 5′-GGATTCGCCATTTTATTTTTCTTG-3′ and 5′-GTATGTATGTGGGTGGGTGTGTCT-3′); TFAM (Q00059, 5′-CTTATAGGGCGGAGTGGCAG-3′ and 5′-GCTGAACGAGGTCTTTTTGGT-3′); NRF1 (Q16656, 5′-CACAGAAAAGGTGCTCAAAGGA-3′ and 5′-TTTGGGTCACTCCGTGTTCC-3′); CytC (P08574, 5′-GAGATGTTCATGCGGCCAG-3′ and 5′-ACGTAGTCCTCACCACCAT-3′); IL1α (P01583, 5′-TCAGCAAAGAAGTCAAGATGGC-3′ and 5′-CCTTCCCGTTGGTTGCTACT-3′); IL1β (P01584, 5′-CTTCGAGGCACAAGGCACAA-3′ and 5′-CTGGAAGGAGCACTTCATCTGT-3′); and IFNγ (P01579, 5′-AGCTCTGCATCGTTTTGGGT-3′ and 5′-TTCTGTCACTCTCCTCTTTCCA-3′). Samples were normalized relative to 18S rRNA (X03205) which served as endogenous control (primer used: 5´-GCCGCTAGAGGTGAAATTCTTG-3´ and 5´-CATTCTTGGCAAATGCTTTCG-3´).

### Analysis of SIRT4 expression in human skin samples

Approval had been obtained from the Ethics Committee of the Heinrich-Heine-University. The study has been conducted according to the ethical rules stated in the Declaration of Helsinki Principles and the ICH GCP guideline was observed insofar as applicable. Healthy human volunteers (n=51) from two age groups (young 18-25 years, and old 60-66 years) were enrolled after written informed consent. All individuals were non-smokers and had no history of any severe skin disease, especially no photosensitivity disorders. Skin types ranged from Fitzpatrick type I to IV. In each volunteer, one 4 mm punch biopsy was taken from neck (i.e. a skin side chronically exposed to UV radiation) and from buttock skin (i.e. a skin side chronically protected from UV radiation), as previously described [98]. Total RNA from human biopsies was isolated as described [99]. Alternatively, skin tissue was disrupted and homogenized in 600 μl RLT+ buffer (+ 1% β-mercaptoethanol) for 60-90 seconds using an Ultra-Turrax.

Primer for SIRT4 [35] were derived from the sequence NM_012240.2 (5´-GCACACTGGGCTTTGAGC-3´ and 5´-CACAATCCAAGCACAGGA-3´). Samples were normalized relative to 18S rRNA (X03205) which served as endogenous control (primer used: 5´-GCCGCTAGAGGTGAAATTCTTG-3´ and 5´-CATTCTTGGCAAATGCTTTCG-3´). For comparison of relative expression in real time PCR of unexposed and exposed skin the 2^−▵▵Ct^ method was used [100]. Alternatively, analysis of SIRT4 mRNA levels in human skin samples was also performed essentially as described above for the analysis of cultured cells.

### Quantification of copy numbers of mir15b and SIRT4 in the epidermis and dermis of human skin

Enzymatic separation of epidermis and dermis: 4-mm skin biopsies were enzymatically separated by incubation with 0.6 mg/ml dispase II (Life Technologies GmbH, Darmstadt, Germany) in PBS without Ca^2+^ and Mg^2+^ (PAA Laboratories GmbH, Cölbe, Germany) for 18 h at 4°C. The epidermal and dermal tissue was homogenized in RLT buffer (Qiagen, Hilden; Germany) using a Mixer Mill (Retsch, Haan, Germany) three times with 26 Hz for 3 min. The lysate was further homogenized using a Qiashredder (Qiagen, Hilden, Germany). Total RNA containing small RNAs was isolated using the RNeasy Mini Kit (Qiagen, Hilden, Germany) following the supplementary protocol “Purification of total RNA containing miRNA from animal cells using the RNeasy Plus Mini Kit”. Additionally, buffer RW1 was replaced by buffer RWT. The RNA concentration was determined by spectrophotometric absorbance at 260 nm.

Real-time quantitative RT-PCR analysis: For quantification of miR-15b diluted RNA was reversely transcribed using universal cDNA synthesis kit (Exiqon, Vedbaek, Denmark). qRT-PCR was performed in quadruplet by using LNA™ PCR primer sets (hsa-miR-15b product no 204243), and SYBR^®^Green PCR kit (both Exiqon, Vedbaek, Denmark). cDNA synthesis started with 20 ng RNA in 20 μl qRT-PCR was performed in 15 μl volumes in a Biorad CFX384 real time PCR detection system using the following settings: 95°C, 10 min; 40 amplification cycle at 95°C (10 s) and 60°C (1 min), 1.6°C/s ramp-rate. For quantification of SIRT4, 50 ng RNA was employed for cDNA-synthesis with M-MLV (Life Technologies GmbH, Darmstadt, Germany). For real time PCR of SIRT4 a TaqMan assay (FAM, # 4331182, Life Technologies GmbH, Darmstadt, Germany) with the following settings was used: 95°C, 10 min; 40 amplification cycles at 95°C (15 s) and 60°C (1 min).

To avoid different expression of housekeeping genes in the two skin compartments, copy numbers were measured as described [101] using a standard DNA fragment which has been purified with a NucleoSpin® (Macherey & Nagel, Düren, Germany). After spectro-photometrical quantification of the fragment the concentration in copy number/biopsy was calculated using the molecular weight of the standard DNA.

### Inhibition of basal and senescence-associated SIRT4 expression in MCF7 cells and primary human dermal fibroblasts by transfection of miR-15b specific “mimics”

Six nmoles of control miRNA mimics or oligonucleotides mimicking endogenous miR-15b function (“miR-15b mimics”; Qiagen, Hilden) were transfected into MCF7 cells seeded on 6-well plates using the HiPerFect transfection reagent (Qiagen) essentially as described by the provider. Immediately thereafter premature senescence by cellular TACC3 depletion was triggered by the addition of doxycycline [22]. SIRT4 mRNA levels were determined four days later by qRT-PCR as described above.

In the case of primary human dermal fibroblasts, cells were transfected with 50 nmoles of control mimics or miR-15b mimics using the DharmaFect transfection reagent essentially as described by the manufacturer (ThermoScientific). Cells were trypsinized twenty four hours after transfection, exposed to γIR (20 Gy) as described above, and thereafter re-seeded on 6-well plates at densities of 7.3×10^3^/cm^2^ (controls) and 10.5×10^3^/cm^2^ (γIR). SIRT4 mRNA levels were determined twenty four hours later by quantitative RT-PCR as described above.

### Immunofluorescence and confocal laser scanning microscopy

Human dermal fibroblasts were subjected to γ-irradiation as described above and seeded in 6-well plates on coverslips. Alternatively, cells were transfected with miR-15b inhibitors in the absence or presence of siRNA duplexes against SIRT4 as described below. Cells were fixed four days later in 4% paraformaldehyde for 20 min and permeabilized with 0.2% Triton X-100 for 20 min followed by a blocking step with 4% BSA/0.05% saponin for 30 min at room temperature. Subsequently, cells were costained with primary antibodies against the mitochondrial marker MTCO2 (abcam, ab3298; 1:500), SIRT4 (Santa Cruz Biotechnology, Inc., sc-135053; 1:200), and α-Tubulin (Acris antibodies, SM568P, 1:500) overnight at 4°C. Secondary antibodies (Alexa Fluor 488-conjugated goat anti-mouse IgG, Alexa Fluor 543-conjugated goat anti-rabbit IgG, and Alexa Fluor 633-conjugated goat anti-rat IgG) were from Life Technologies and used at a dilution of 1:500 for 1 hr at room temperature. Analyses were performed with a LSM510-Meta confocal microscope (Zeiss) equipped with 40/1.3 or 63/1.4 immersion objectives and excitation wavelengths of 488 nm, 543 nm, and 633 nm. Cells were examined and pictures were taken at the Z-stack level (0.5 μm) and when indicated further analysed using the ImageJ software v1.49k as described in the suppl. Materials & Methods part.

### Transfection of miR-15b “inhibitors” and siRNA duplexes to modulate SIRT4 expression

Primary human dermal fibroblasts were transfected in six-well plates (2×10^5^ cells/well) with 6.5 nmoles of miR-15b “inhibitor” oligos (Qiagen, MIN0004586) or control oligos (Qiagen, 1022076) in the presence or absence of 10 nmoles of 27-mer siRNA duplexes against human SIRT4 (Origene, SR308254) using the DharmaFect transfection reagent essentially as described above. Cells were analysed two, three, and four days after transfection. Transfection efficiency was controlled by employing the siGLO Red transfection indicator (GE Healthcare, D-001630-02-05).

### Measurement of mitochondrial reactive oxygen species (ROS) and mitochondrial membrane potential

Cells were costained with 100 nM MitoTrackerGreen® (Cell Signaling Technology, #9074) and 5 μM MitoSoxRed™ mitochondrial superoxide indicator (Life Technologies, M36008) to determine mitochondrial ROS levels. JC-1 (2 μM; Life Technologies, T3168) was employed to determine the mitochondrial membrane potential. Protocols were followed and flow cytometric measurements performed on a FACSCalibur™ (BD) essentially as described [102]. MitoQ [10-(6′-ubiquinonyl) decyltriphenyl-phosphonium bromide] [72] (kindly provided by Dr. Mike Murphy, Cambridge) was used as a mitochondria-specific antioxidant thereby treating fibroblasts with a concentration of 100 nM for three days.

### Analysis of mitochondrial morphology and fragmentation

Human dermal fibroblasts were transfected with miR-15b inhibitors in the presence or absence of siRNA duplexes against SIRT4 as described above. Four days later, cells were fixed and stained with SIRT4 and MTCO2 specific antibodies. The mitochondrial network was analysed as described [103] employing an ImageJ Mito-Morphology macro (http://imagejdocu.tudor.lu/doku.php?id=plugin:morphology:mitochondrial_morphology_macro_plug-in:start) [104] to evaluate microscopic pictures taken by confocal laser scanning microscopy. Cellular morphology/size was defined by parallel α-Tubulin staining.

### Statistical analysis

*In-vitro* data are typically presented as mean ± SD, *in-vivo* data as vertical point plot with median indicated as horizontal line. When comparing two groups, paired, unpaired t-test, or the non-parametric Wilcoxon signed rank or Mann-Whitney rank sum tests were employed. Multiple comparisons were analysed by one-way analysis of variance (ANOVA) (or on ranks) followed by SNK.

## SUPPLEMENTARY MATERIALS AND METHODS, TABLES, FIGURES AND DATASETS







## Abbreviations

CytC: cytochrom C
γIR: gamma-irradiation
GDH: glutamate dehydrogenase
miRNA: microRNA
mitoQ: 10-(6′-ubiquinonyl) decyltriphenylphosphonium bromide
MMP1: matrix metalloproteinase-1
MTCO2: mitochondrially encoded cytochrome C oxidase II
mtSIRT: mitochondrially localized sirtuin
NRF1: nuclear respiratory factor 1
PDH: pyruvate dehydrogenase
qRT-PCR: quantitative real-time PCR
ROS: reactive oxygen species
SASP: senescence associated secretory phenotype
SIRT: sirtuin
TACC3: Transforming Acidic Coiled Coil 3
TFAM: transcription factor A
UTR: untranslated region
UV: ultraviolet
VEGF: vascular endothelial growth factor

## References

[1] Krutmann J, Gilchrest BA, Gilchrest BA, Krutmann J (2006). Photoaging of skin. Skin Aging.

[2] Fisher GJ, Wang ZQ, Datta SC, Varani J, Kang S, Voorhees JJ (1997). Pathophysiology of premature skin aging induced by ultraviolet light. N Engl J Med.

[3] Krutmann J, Schroeder P (2009). Role of mitochondria in photoaging of human skin: the defective powerhouse model. J Investig Dermatol Symp Proc.

[4] Koziel R, Greussing R, Maier AB, Declercq L, Jansen-Durr P (2011). Functional interplay between mitochondrial and proteasome activity in skin aging. J Invest Dermatol.

[5] Tigges J, Krutmann J, Fritsche E, Haendeler J, Schaal H, Fischer JW, Kalfalah F, Reinke H, Reifenberger G, Stuhler K, Ventura N, Gundermann S, Boukamp P (2014). The hallmarks of fibroblast ageing. Mech Ageing Dev.

[6] Campisi J, d'Adda di Fagagna F (2007). Cellular senescence: when bad things happen to good cells. Nat Rev Mol Cell Biol.

[7] Ben-Porath I, Weinberg RA (2005). The signals and pathways activating cellular senescence. Int J Biochem Cell Biol.

[8] Collado M, Serrano M (2006). The power and the promise of oncogene-induced senescence markers. Nat Rev Cancer.

[9] Moiseeva O, Bourdeau V, Roux A, Deschenes-Simard X, Ferbeyre G (2009). Mitochondrial dysfunction contributes to oncogene-induced senescence. Mol Cell Biol.

[10] Passos JF, Nelson G, Wang C, Richter T, Simillion C, Proctor CJ, Miwa S, Olijslagers S, Hallinan J, Wipat A, Saretzki G, Rudolph KL, Kirkwood TB (2010). Feedback between p21 and reactive oxygen production is necessary for cell senescence. Mol Syst Biol.

[11] Passos JF, Zglinicki TV (2012). Mitochondrial dysfunction and cell senescence - skin deep into mammalian aging. Aging (Albany NY).

[12] Chuaire-Noack L (2010). The dual role of senescence in tumorigenesis. Int J Morphol.

[13] Hildebrand DG, Lehle S, Borst A, Haferkamp S, Essmann F, Schulze-Osthoff K (2013). alpha-Fucosidase as a novel convenient biomarker for cellular senescence. Cell Cycle.

[14] Singh M, Piekorz RP (2013). Senescence-associated lysosomal alpha-L-fucosidase (SA-alpha-Fuc): A sensitive and more robust biomarker for cellular senescence beyond SA-beta-Gal. Cell Cycle.

[15] Campisi J (2013). Aging, cellular senescence, and cancer. Annu Rev Physiol.

[16] Demaria M, Desprez PY, Campisi J, Velarde MC (2015). Cell autonomous and non-autonomous effects of senescent cells in the skin. J Invest Dermatol.

[17] Rodier F, Campisi J (2011). Four faces of cellular senescence. J Cell Biol.

[18] Waldera-Lupa DM, Kalfalah F, Safferling K, Boukamp P, Poschmann G, Volpi E, Gotz-Rosch C, Bernerd F, Haag L, Huebenthal U, Fritsche E, Boege F, Grabe N (2015). Characterization of skin aging associated secreted proteins (SAASP) produced by dermal fibroblasts isolated from intrinsically aged human skin. J Invest Dermatol.

[19] Schmidt S, Essmann F, Cirstea IC, Kuck F, Thakur HC, Singh M, Kletke A, Janicke RU, Wiek C, Hanenberg H, Ahmadian MR, Schulze-Osthoff K, Nurnberg B (2010). The centrosome and mitotic spindle apparatus in cancer and senescence. Cell Cycle.

[20] Thakur HC, Singh M, Nagel-Steger L, Kremer J, Prumbaum D, Fansa EK, Ezzahoini H, Nouri K, Gremer L, Abts A, Schmitt L, Raunser S, Ahmadian MR (2014). The centrosomal adaptor TACC3 and the microtubule polymerase chTOG interact via defined C-terminal subdomains in an Aurora-A kinase-independent manner. J Biol Chem.

[21] Thakur HC, Singh M, Nagel-Steger L, Prumbaum D, Kalawy Fansa E, Gremer L, Ezzahoini H, Abts A, Schmitt L, Raunser S, Ahmadian MR, Piekorz RP (2013). Role of centrosomal adaptor proteins of the TACC family in the regulation of microtubule dynamics during mitotic cell division. Biol Chem.

[22] Schmidt S, Schneider L, Essmann F, Cirstea IC, Kuck F, Kletke A, Jänicke RU, Wiek C, Hanenberg H, Ahmadian MR, Schulze-Osthoff K, Nurnberg B, Piekorz RP (2010). The centrosomal protein TACC3 controls paclitaxel sensitivity by modulating a premature senescence program. Oncogene.

[23] Haigis MC, Sinclair DA (2010). Mammalian sirtuins: biological insights and disease relevance. Annu Rev Pathol.

[24] Michishita E, Park JY, Burneskis JM, Barrett JC, Horikawa I (2005). Evolutionarily conserved and nonconserved cellular localizations and functions of human SIRT proteins. Mol Biol Cell.

[25] Benavente CA, Schnell SA, Jacobson EL (2012). Effects of niacin restriction on sirtuin and PARP responses to photodamage in human skin. PLoS One.

[26] Chang HC, Guarente L (2014). SIRT1 and other sirtuins in metabolism. Trends Endocrinol Metab.

[27] Donmez G, Guarente L (2010). Aging and disease: connections to sirtuins. Aging Cell.

[28] Guarente L (2007). Sirtuins in aging and disease. Cold Spring Harb Symp Quant Biol.

[29] Palacios JA, Herranz D, De Bonis ML, Velasco S, Serrano M, Blasco MA (2010). SIRT1 contributes to telomere maintenance and augments global homologous recombination. J Cell Biol.

[30] Sasaki T, Maier B, Bartke A, Scrable H (2006). Progressive loss of SIRT1 with cell cycle withdrawal. Aging Cell.

[31] German NJ, Haigis MC (2015). Sirtuins and the Metabolic Hurdles in Cancer. Curr Biol.

[32] Li X, Kazgan N (2011). Mammalian sirtuins and energy metabolism. Int J Biol Sci.

[33] Pirinen E, Lo Sasso G, Auwerx J (2012). Mitochondrial sirtuins and metabolic homeostasis. Best Pract Res Clin Endocrinol Metab.

[34] Verdin E, Hirschey MD, Finley LW, Haigis MC (2010). Sirtuin regulation of mitochondria: energy production, apoptosis, and signaling. Trends Biochem Sci.

[35] Haigis MC, Mostoslavsky R, Haigis KM, Fahie K, Christodoulou DC, Murphy AJ, Valenzuela DM, Yancopoulos GD, Karow M, Blander G, Wolberger C, Prolla TA, Weindruch R (2006). SIRT4 inhibits glutamate dehydrogenase and opposes the effects of calorie restriction in pancreatic beta cells. Cell.

[36] Laurent G, German NJ, Saha AK, de Boer VC, Davies M, Koves TR, Dephoure N, Fischer F, Boanca G, Vaitheesvaran B, Lovitch SB, Sharpe AH, Kurland IJ (2013). SIRT4 coordinates the balance between lipid synthesis and catabolism by repressing malonyl CoA decarboxylase. Mol Cell.

[37] Mathias RA, Greco TM, Oberstein A, Budayeva HG, Chakrabarti R, Rowland EA, Kang Y, Shenk T, Cristea IM (2014). Sirtuin 4 is a lipoamidase regulating pyruvate dehydrogenase complex activity. Cell.

[38] Chainiaux F, Magalhaes JP, Eliaers F, Remacle J, Toussaint O (2002). UVB-induced premature senescence of human diploid skin fibroblasts. Int J Biochem Cell Biol.

[39] Debacq-Chainiaux F, Borlon C, Pascal T, Royer V, Eliaers F, Ninane N, Carrard G, Friguet B, de Longueville F, Boffe S, Remacle J, Toussaint O (2005). Repeated exposure of human skin fibroblasts to UVB at subcytotoxic level triggers premature senescence through the TGF-beta1 signaling pathway. J Cell Sci.

[40] Essmann F, Engels IH, Totzke G, Schulze-Osthoff K, Janicke RU (2004). Apoptosis resistance of MCF-7 breast carcinoma cells to ionizing radiation is independent of p53 and cell cycle control but caused by the lack of caspase-3 and a caffeine-inhibitable event. Cancer Res.

[41] Bluwstein A, Kumar N, Leger K, Traenkle J, Oostrum J, Rehrauer H, Baudis M, Hottiger MO (2013). PKC signaling prevents irradiation-induced apoptosis of primary human fibroblasts. Cell Death Dis.

[42] Coppe JP, Desprez PY, Krtolica A, Campisi J (2010). The senescence-associated secretory phenotype: the dark side of tumor suppression. Annu Rev Pathol.

[43] Garbers C, Kuck F, Aparicio-Siegmund S, Konzak K, Kessenbrock M, Sommerfeld A, Haussinger D, Lang PA, Brenner D, Mak TW, Rose-John S, Essmann F, Schulze-Osthoff K (2013). Cellular senescence or EGFR signaling induces Interleukin 6 (IL-6) receptor expression controlled by mammalian target of rapamycin (mTOR). Cell Cycle.

[44] Dulic V (2013). Senescence regulation by mTOR. Methods Mol Biol.

[45] Csibi A, Fendt SM, Li C, Poulogiannis G, Choo AY, Chapski DJ, Jeong SM, Dempsey JM, Parkhitko A, Morrison T, Henske EP, Haigis MC, Cantley LC (2013). The mTORC1 pathway stimulates glutamine metabolism and cell proliferation by repressing SIRT4. Cell.

[46] Bhaumik D, Scott GK, Schokrpur S, Patil CK, Orjalo AV, Rodier F, Lithgow GJ, Campisi J (2009). MicroRNAs miR-146a/b negatively modulate the senescence-associated inflammatory mediators IL-6 and IL. Aging (Albany NY).

[47] Greussing R, Hackl M, Charoentong P, Pauck A, Monteforte R, Cavinato M, Hofer E, Scheideler M, Neuhaus M, Micutkova L, Mueck C, Trajanoski Z, Grillari J (2013). Identification of microRNA-mRNA functional interactions in UVB-induced senescence of human diploid fibroblasts. BMC Genomics.

[48] Hackl M, Brunner S, Fortschegger K, Schreiner C, Micutkova L, Muck C, Laschober GT, Lepperdinger G, Sampson N, Berger P, Herndler-Brandstetter D, Wieser M, Kuhnel H (2010). miR-17, miR-19b, miR-20a, and miR-106a are down-regulated in human aging. Aging Cell.

[49] Holly AC, Grellscheid S, van de Walle P, Dolan D, Pilling LC, Daniels DJ, von Zglinicki T, Ferrucci L, Melzer D, Harries LW (2015). Comparison of senescence-associated miRNAs in primary skin and lung fibroblasts. Biogerontology.

[50] Hong L, Lai M, Chen M, Xie C, Liao R, Kang YJ, Xiao C, Hu WY, Han J, Sun P (2010). The miR-17-92 Cluster of microRNAs Confers Tumorigenicity by Inhibiting Oncogene-Induced Senescence. Cancer Res.

[51] Lafferty-Whyte K, Cairney CJ, Jamieson NB, Oien KA, Keith WN (2009). Pathway analysis of senescence-associated miRNA targets reveals common processes to different senescence induction mechanisms. Biochim Biophys Acta.

[52] Grillari J, Hackl M, Grillari-Voglauer R (2010). miR-17-92 cluster: ups and downs in cancer and aging. Biogerontology.

[53] Xu N, Brodin P, Wei T, Meisgen F, Eidsmo L, Nagy N, Kemeny L, Stahle M, Sonkoly E, Pivarcsi A (2011). MiR-125b, a microRNA downregulated in psoriasis, modulates keratinocyte proliferation by targeting FGFR2. J Invest Dermatol.

[54] Xu D, Takeshita F, Hino Y, Fukunaga S, Kudo Y, Tamaki A, Matsunaga J, Takahashi RU, Takata T, Shimamoto A, Ochiya T, Tahara H (2011). miR-22 represses cancer progression by inducing cellular senescence. J Cell Biol.

[55] Faraonio R, Salerno P, Passaro F, Sedia C, Iaccio A, Bellelli R, Nappi TC, Comegna M, Romano S, Salvatore G, Santoro M, Cimino F (2012). A set of miRNAs participates in the cellular senescence program in human diploid fibroblasts. Cell Death Differ.

[56] Marasa BS, Srikantan S, Masuda K, Abdelmohsen K, Kuwano Y, Yang X, Martindale JL, Rinker-Schaeffer CW, Gorospe M (2009). Increased MKK4 abundance with replicative senescence is linked to the joint reduction of multiple microRNAs. Sci Signal.

[57] Vierkotter A, Ranft U, Kramer U, Sugiri D, Reimann V, Krutmann J (2009). The SCINEXA: a novel, validated score to simultaneously assess and differentiate between intrinsic and extrinsic skin ageing. J Dermatol Sci.

[58] Ning MS, Andl T (2013). Control by a hair's breadth: the role of microRNAs in the skin. Cell Mol Life Sci.

[59] Huang P, Bi J, Owen GR, Chen W, Rokka A, Koivisto L, Heino J, Hakkinen L, Larjava H (2015). Keratinocyte Microvesicles Regulate the Expression of Multiple Genes in Dermal Fibroblasts. J Invest Dermatol.

[60] Dong K, Pelle E, Yarosh DB, Pernodet N (2012). Sirtuin 4 identification in normal human epidermal keratinocytes and its relation to sirtuin 3 and energy metabolism under normal conditions and UVB-induced stress. Exp Dermatol.

[61] Lasserre C, D'Arcangelis A, Mildner M, Bhatt P, Tschachler E (2007). The effect of ultraviolet irradiation on sirtuin expression in human skin. J Invest Dermatol.

[62] Serravallo M, Jagdeo J, Glick SA, Siegel DM, Brody NI (2013). Sirtuins in dermatology: applications for future research and therapeutics. Arch Dermatol Res.

[63] Finnerty JR, Wang WX, Hebert SS, Wilfred BR, Mao G, Nelson PT (2010). The miR-15/107 group of microRNA genes: evolutionary biology, cellular functions, and roles in human diseases. J Mol Biol.

[64] Maes OC, Sarojini H, Wang E (2009). Stepwise up-regulation of microRNA expression levels from replicating to reversible and irreversible growth arrest states in WI-38 human fibroblasts. J Cell Physiol.

[65] Satzger I, Mattern A, Kuettler U, Weinspach D, Voelker B, Kapp A, Gutzmer R (2010). MicroRNA-15b represents an independent prognostic parameter and is correlated with tumor cell proliferation and apoptosis in malignant melanoma. Int J Cancer.

[66] Bueno MJ, Gomez de Cedron M, Laresgoiti U, Fernandez-Piqueras J, Zubiaga AM, Malumbres M (2010). Multiple E2F-induced microRNAs prevent replicative stress in response to mitogenic signaling. Mol Cell Biol.

[67] Ofir M, Hacohen D, Ginsberg D (2011). miR-15 and miR-16 are direct transcriptional targets of E2F1 that limit E2F-induced proliferation by targeting Cyclin E. Mol Cancer Res.

[68] Chen T, Xue L, Niu J, Ma L, Li N, Cao X, Li Q, Wang M, Zhao W, Li G, Wang J, Tong T (2012). The retinoblastoma protein selectively represses E2F1 targets via a TAAC DNA element during cellular senescence. J Biol Chem.

[69] He S, Yang S, Deng G, Liu M, Zhu H, Zhang W, Yan S, Quan L, Bai J, Xu N (2010). Aurora kinase A induces miR-17-92 cluster through regulation of E2F1 transcription factor. Cell Mol Life Sci.

[70] Nishi H, Ono K, Iwanaga Y, Horie T, Nagao K, Takemura G, Kinoshita M, Kuwabara Y, Mori RT, Hasegawa K, Kita T, Kimura T (2010). MicroRNA-15b modulates cellular ATP levels and degenerates mitochondria via Arl2 in neonatal rat cardiac myocytes. J Biol Chem.

[71] Li N, Bates DJ, An J, Terry DA, Wang E (2009). Up-regulation of key microRNAs, and inverse down-regulation of their predicted oxidative phosphorylation target genes, during aging in mouse brain. Neurobiol Aging.

[72] Saretzki G, Murphy MP, von Zglinicki T (2003). MitoQ counteracts telomere shortening and elongates lifespan of fibroblasts under mild oxidative stress. Aging Cell.

[73] Verma M, Shulga N, Pastorino JG (2013). Sirtuin-4 modulates sensitivity to induction of the mitochondrial permeability transition pore. Biochim Biophys Acta.

[74] Zorov DB, Juhaszova M, Sollott SJ (2014). Mitochondrial reactive oxygen species (ROS) and ROS-induced ROS release. Physiol Rev.

[75] Stacpoole PW (2012). The pyruvate dehydrogenase complex as a therapeutic target for age-related diseases. Aging Cell.

[76] Fan X, Hussien R, Brooks GA (2010). H2O2-induced mitochondrial fragmentation in C2C12 myocytes. Free Radic Biol Med.

[77] Willems PH, Rossignol R, Dieteren CE, Murphy MP, Koopman WJ (2015). Redox Homeostasis and Mitochondrial Dynamics. Cell Metab.

[78] de Moura MB, Uppala R, Zhang Y, Van Houten B, Goetzman ES (2014). Overexpression of mitochondrial sirtuins alters glycolysis and mitochondrial function in HEK293 cells. PLoS One.

[79] Ho L, Titus AS, Banerjee KK, George S, Lin W, Deota S, Saha AK, Nakamura K, Gut P, Verdin E, Kolthur-Seetharam U (2013). SIRT4 regulates ATP homeostasis and mediates a retrograde signaling via AMPK. Aging (Albany NY).

[80] Shimazu T, Hirschey MD, Hua L, Dittenhafer-Reed KE, Schwer B, Lombard DB, Li Y, Bunkenborg J, Alt FW, Denu JM, Jacobson MP, Verdin E (2010). SIRT3 deacetylates mitochondrial 3-Hydroxy-3-Methylglutaryl CoA synthase 2 and regulates ketone body production. Cell Metab.

[81] Kim HS, Patel K, Muldoon-Jacobs K, Bisht KS, Aykin-Burns N, Pennington JD, van der Meer R, Nguyen P, Savage J, Owens KM, Vassilopoulos A, Ozden O, Park SH (2010). SIRT3 is a mitochondria-localized tumor suppressor required for maintenance of mitochondrial integrity and metabolism during stress. Cancer Cell.

[82] Hirschey MD, Shimazu T, Jing E, Grueter CA, Collins AM, Aouizerat B, Stancakova A, Goetzman E, Lam MM, Schwer B, Stevens RD, Muehlbauer MJ, Kakar S (2011). SIRT3 deficiency and mitochondrial protein hyperacetylation accelerate the development of the metabolic syndrome. Mol Cell.

[83] Dittenhafer-Reed KE, Richards AL, Fan J, Smallegan MJ, Fotuhi Siahpirani A, Kemmerer ZA, Prolla TA, Roy S, Coon JJ, Denu JM (2015). SIRT3 mediates multi-tissue coupling for metabolic fuel switching. Cell Metab.

[84] Nakagawa T, Lomb DJ, Haigis MC, Guarente L (2009). SIRT5 Deacetylates carbamoyl phosphate synthetase 1 and regulates the urea cycle. Cell.

[85] Nishida Y, Rardin MJ, Carrico C, He W, Sahu AK, Gut P, Najjar R, Fitch M, Hellerstein M, Gibson BW, Verdin E (2015). SIRT5 Regulates both Cytosolic and Mitochondrial Protein Malonylation with Glycolysis as a Major Target. Mol Cell.

[86] Jeong SM, Lee A, Lee J, Haigis MC (2014). SIRT4 protein suppresses tumor formation in genetic models of Myc-induced B cell lymphoma. J Biol Chem.

[87] Jeong SM, Xiao C, Finley LW, Lahusen T, Souza AL, Pierce K, Li YH, Wang X, Laurent G, German NJ, Xu X, Li C, Wang RH (2013). SIRT4 has tumor-suppressive activity and regulates the cellular metabolic response to DNA damage by inhibiting mitochondrial glutamine metabolism. Cancer Cell.

[88] Nasrin N, Wu X, Fortier E, Feng Y, Bare OC, Chen S, Ren X, Wu Z, Streeper RS, Bordone L (2010). SIRT4 regulates fatty acid oxidation and mitochondrial gene expression in liver and muscle cells. J Biol Chem.

[89] Campbell CT, Kolesar JE, Kaufman BA (2012). Mitochondrial transcription factor A regulates mitochondrial transcription initiation, DNA packaging, and genome copy number. Biochim Biophys Acta.

[90] Hock MB, Kralli A (2009). Transcriptional control of mitochondrial biogenesis and function. Annu Rev Physiol.

[91] Biswas M, Chan JY (2010). Role of Nrf1 in antioxidant response element-mediated gene expression and beyond. Toxicol Appl Pharmacol.

[92] Cahu J (2013). SASP: roadblock for tissue re-organization. Aging (Albany NY).

[93] Coleman PR, Chang G, Hutas G, Grimshaw M, Vadas MA, Gamble JR (2013). Age-associated stresses induce an anti-inflammatory senescent phenotype in endothelial cells. Aging (Albany NY).

[94] Waldera-Lupa DM, Kalfalah F, Safferling K, Boukamp P, Poschmann G, Volpi E, Gotz-Rosch C, Bernerd F, Haag L, Huebenthal U, Fritsche E, Boege F, Grabe N (2015). Characterization of Skin aging Associated Secreted Proteins (SAASP) Produced by Dermal Fibroblasts Isolated from Intrinsically Aged Human Skin. J Invest Dermatol.

[95] Zhong G, Cheng X, Long H, He L, Qi W, Xiang T, Zhao Z, Zhu B (2013). Dynamically expressed microRNA-15b modulates the activities of CD8+ T lymphocytes in mice with Lewis lung carcinoma. J Transl Med.

[96] Freund A, Patil CK, Campisi J (2011). p38MAPK is a novel DNA damage response-independent regulator of the senescence-associated secretory phenotype. EMBO J.

[97] Schmittgen TD, Livak KJ (2008). Analyzing real-time PCR data by the comparative C(T) method. Nat Protoc.

[98] Berneburg M, Gattermann N, Stege H, Grewe M, Vogelsang K, Ruzicka T, Krutmann J (1997). Chronically ultraviolet-exposed human skin shows a higher mutation frequency of mitochondrial DNA as compared to unexposed skin and the hematopoietic system. Photochem Photobiol.

[99] Grether-Beck S, Muhlberg K, Brenden H, Felsner I, Brynjolfsdottir A, Einarsson S, Krutmann J (2008). Bioactive molecules from the Blue Lagoon: in vitro and in vivo assessment of silica mud and microalgae extracts for their effects on skin barrier function and prevention of skin ageing. Exp Dermatol.

[100] Livak KJ, Schmittgen TD (2001). Analysis of relative gene expression data using real-time quantitative PCR and the 2(−Delta Delta C(T)) Method. Methods.

[101] Grether-Beck S, Felsner I, Brenden H, Kohne Z, Majora M, Marini A, Jaenicke T, Rodriguez-Martin M, Trullas C, Hupe M, Elias PM, Krutmann J (2012). Urea uptake enhances barrier function and antimicrobial defense in humans by regulating epidermal gene expression. J Invest Dermatol.

[102] Kalfalah F, Sobek S, Bornholz B, Gotz-Rosch C, Tigges J, Fritsche E, Krutmann J, Kohrer K, Deenen R, Ohse S, Boerries M, Busch H, Boege F (2014). Inadequate mito-biogenesis in primary dermal fibroblasts from old humans is associated with impairment of PGC1A-independent stimulation. Exp Gerontol.

[103] Barbato DL, Tatulli G, Aquilano K, Ciriolo MR (2015). Mitochondrial Hormesis links nutrient restriction to improved metabolism in fat cell. Aging (Albany NY).

[104] Dagda RK, Cherra SJ, Kulich SM, Tandon A, Park D, Chu CT (2009). Loss of PINK1 function promotes mitophagy through effects on oxidative stress and mitochondrial fission. J Biol Chem.

