# Supplementary material for: MicroRNA-15b regulates mitochondrial ROS production and the senescence-associated secretory phenotype through sirtuin 4/SIRT4
Source: Aging (Albany NY). 2016 Feb 26;8(3):484–505. doi: 10.18632/aging.100905 (PMC4833141; doi:10.18632/aging.100905)
Supplement: Supplementary file 1 [file aging-08-484-s001.pdf]

SUPPLEMENTARY MATERIALS AND METHODS

Cellular TACC3 depletion in MCF7 cells. The centrosomal protein TACC3 was depleted essentially as described [1-3]. Cells were plated at a density of $3x10^3/cm^2$ (control shRNA) and $6.7x10^3/cm^2$ (TACC3 shRNA). Expression of control or TACC3 shRNA was induced by adding doxycycline (5 µg/ml) to the culture medium for 4 days.

Ionizing γ-irradiation (γIR) of MCF7 cells. Trypsinized cells were exposed to γIR (20 Gy) using a Gammacell 1000 Elite irradiator (Nordion International, Inc., Fleurus, Belgium). After irradiation, MCF7 cells were plated at a density of $6.7x10^3/cm^2$, $3.3x10^3/cm^2$, $0.8x10^3/cm^2$ (control), and $8.3x10^3/cm^2$ (20 Gy) and harvested for analysis on days 2, 4 and 6 after treatment.

Repetitive UV treatment of primary human skin fibroblasts. Human dermal fibroblasts were isolated from the foreskin of four different donors (age: 1 to 3 years). Approval had been obtained from the Ethics Committee of the Heinrich-Heine-University. The study has been conducted according to the ethical rules stated in the Declaration of Helsinki Principles and the ICH GCP guideline was followed as applicable. Cells were cultivated in Eagle's minimum essential medium with Earle's salts (MEM, PAA Laboratories, Pasching, Austria) and supplemented with 10% fetal bovine serum (Invitrogen, Karlsruhe, Germany), 1% antibiotics/ antimycotics (penicillin, streptomycin, amphotericin B), and 1% glutamine (Invitrogen) and then cultivated on 100 mm plastic culture dishes (Greiner, Solingen, Germany) at 37°C in humidified air with 5% CO_2. Cells were used between passages five and ten and grown to confluency before treatment. Twenty four hours before UV irradiation media were changed to serum-free MEM.

Cells were exposed once daily to a single dose of 100 mW/m² ultraviolet radiation from TL20W/12RS SLV (Phillips, Hamburg, Germany) fluorescent bulbs, which primarily emit in the range of 290-315 nm (= UVB) for a period of five days. For irradiation, medium was replaced by phosphate-buffered saline (37°C, Invitrogen). Control cells (sham treated) were held on room temperature under similar conditions, but without irradiation. Following the last treatment, cells were cultivated for 24 and 72 hours with serum-free MEM medium at 37°C.

Quantification of copy numbers of miR-15/16 family members in primary human dermal fibroblasts exposed to γIR. Isolation of miRNAs from control and γIR-treated (20 Gy) fibroblasts and real-time quantitative RT-PCR analysis was performed essentially as described in the Material and Methods section of the manuscript. PCR primer sets miRCURY LNA™ Universal RT were obtained from Exiqon (Vedbaek, Denmark) and used were: hsa-miR-15b, product no. 204243; miR-15a, product no. 204066; miR-16-1, product no. 205902; miR-16-2 product no. 204309; miR-195, product no. 204128; miR-497, product no. 204354.

Quantification of SIRT4 protein levels by ELISA. Cells were lysed in buffer containing 1% Nonidet P-40, 50 mM Tris/HCl, pH 8.0, 150 mM NaCl, and 2 mM EDTA and protein concentrations were measured using the Bio-Rad protein assay kit. Ninety-six well plates (M9410, Sigma) were coated overnight at 4°C with 20 µg protein lysate in 100 µl coating buffer (0.03 M Na_2CO_3, 0.07 M $NaHCO_3$, pH 9.6). Plates were washed with PBS-T (0.05% Tween) and blocked with 1% BSA for 2 h at room temperature followed by successive incubation steps with a primary antibody against SIRT4 (H-300, Santa Cruz; overnight at 4 °C) diluted in blocking solution (1:200) and secondary antibodies (#7074, Cell Signaling; 1:1000; 2 h at room temperature). Signal development was started by the addition of 100 µl BM Blue POD Substrate (11442066001, Roche), stopped by adding 100 µl H_2SO_4 (1.8 M), and analyzed at 450 nm using an Infinite 200 PRO NanoQuant Microplate Reader (Tecan).

Calculation of the SCINEXA™ Score. Calculation of the SCINEXA™ Score was performed as described previously [4] except for the following adaptations: In this study the different skin parameters were scored from 0 to 5, not 0 to 3. Regarding extrinsic skin aging the following parameters were not included: Yellowness and dryness of the skin, pseudo scars, permanent erythema as well as the pathological skin conditions (carcinomas, melanoma, actinic pre-cancerosis) as these were exclusion criteria of the study. Instead, we included sunburn and lentigines solaris at two different body sites and evaluated coarse wrinkles at six different locations. Regarding intrinsic skin aging we evaluated lax skin appearance at two different body sites instead of judging the overall appearance of the skin. In addition coarse wrinkles formation was evaluated at six different locations while reduced fat tissue was not included as a scoring parameter. The overall number of parameters remained the same as in the original publication.

Analysis of SMC4 and DLEU2 expression in human skin samples. Expression levels of SMC4 and DLEU2 were determined by real-time quantitative RT-PCR

analysis as described in the Material and Methods section of the manuscript. PCR primer for SMC4 and DLEU2 were derived from the sequences:
NM_05496.3 (SMC4: 5´-
TCG AAG CCA TGG AAT TGA CTT-3´ and 5´-
TGT TCA GTC TGG-
CCT TTT GGT-3´) and
NM_002612.1 (DLEU2: 5´-
AGC ACA GTG GAA CTA GAT CCT AGT ACA G-
3´ and 5´-GTT-
TGG AGA TCA GTA GAA GGA AAT TTC-3´).

Microarray analysis (mRNA and miRNA). Microarray analyses were done using the "Quick Amp Labeling Kit, one color" (Agilent Technologies Cat.-No 5190-0442) and the RNA Spike In Kit, one color (Agilent Technologies Cat.-No 5188-5282) following the manufacturer's protocol. We used 500 ng of total RNA as starting material for cDNA synthesis. The cDNA synthesis and *in vitro* transcription (IVT) were performed according the manufacturer's instructions. The quantity and labeling efficiency of the labeled cRNA were measured photometrical (NanoDrop ND-1000, Fisher Scientific). For gene expression analysis the labeled cRNA was hybridized onto "Whole Human Genome 4x44K Microarrays" (Agilent Technologies, Cat.-No G4112F) for 17 hours at 65°C and 10 rpm in the hybridization oven (Agilent Technologies). For miRNA expression analysis the "Human miRNA Microarray (V2)" (Cat.-No G4471A-019118; Agilent Technologies) was employed. Washing of the arrays were performed according to the manufacturer's recommendation. The Cy3 fluorescence intensities were detected by scanning the arrays using the Agilent DNA microarray scanner (G2505B). The resulting image files were analyzed with Feature extraction software. Raw data files were processed and analysed using the GeneSpring Software Version 11 (Agilent Technologies) and were deposited at GEO (Gene Expression Omnibus) (www.ncbi.nlm.nih.gov/geo/query/acc.cgi?acc=GSE45729).

SIRT4 - 3'-UTR luciferase bindings assays employing miR-15b specific "mimics. HeLa cells were grown on 6-well plates and seeded at $1.2x10^5$ cells per well 24 h. before transfection using the lipofectamine 2000 transfection reagent (Life Technologies). Cells were co-transfected with firefly luciferase reporter plasmids (4 µg; pMirTarget, Origene; SC202362; harboring the SIRT4 - 3'-UTR with a wild-type miR-15b seed sequence [5'-TGCTGCTA-3'] or a mutated miR-15b seed sequence [5'-TGC***GA***CTA-3']) and the pRL-SV40 renilla luciferase vector (0.3 µg; Promega) together with 50 nmoles of oligonucleotides mimicking endogenous miR-15b function ("miR-15b mimics"; Qiagen) or control miRNA mimics. Cells were harvested 36 h after transfection and luciferase activity was determined using the Dual Luciferase® assay system (Promega) and a MicrolumatPlus Luminometer LB96V (EG&G Berthold). Depicted are normalized luciferase activities (ratio of firefly/renilla luciferase signal intensities).

Enzymatic glutamate dehydrogenase (GDH) activity assay. GDH activity was determined colorimetrically employing an assay kit (Sigma-Aldrich, MAK099) essentially as described by the manufacturer. Cells were lysed in GDH assay buffer and protein concentration of the lysates was measured using the protein assay kit II (Bio-Rad, #500-0002). Enzymatic activities were determined from 25 µg of lysate by calculating the rates of enzymatic activity from the slope of the linear part of the reaction curve.

Immunoblot analysis. Total cell lysates were prepared in lysis buffer containing 1% Nonidet P-40, 50 mM Tris/HCl, pH 8.0, 150 mM NaCl, and 2 mM EDTA. Protein samples were separated by SDS-PAGE and transferred to nitrocellulose membranes (Hybond C, GE Healthcare, Freiburg, Germany). Blots were probed with an E2F1-specific antibody (#3742; Cell Signaling). Following incubation with the secondary antibody signals were visualized using a LI-COR imaging system. GAPDH levels (antibody from Abcam, Cambridge) served as loading control.

Image analysis of confocal microscopic pictures. Stacked confocal images were analyzed using the ImageJ software v1.49k (Z project average, using the α-Tubulin signal to create a region of interest; filter: median, radius: 4; background substracted, rolling=200; threshold: huang white; particles analyzed from the size of 500 px to infinity, edges were excluded) by employing the following macro command:

```
original = getTitle();
# run Z Project "Median" with all ten pictures
run("Z Project...");
run("Split Channels");
# select the alpha-Tubulin-channel
selectWindow("C4-AVG_" + original);
run("Median...", "radius=4");
run("Subtract Background...", "rolling=200");
run("Auto Threshold", "method=Huang white");
run("Watershed")
run("Analyze Particles...", "size=500-Infinity exclude add");
selectWindow("C2-AVG_" + original);
roiManager("Measure");
close();
selectWindow("C3-AVG_" + original);
roiManager("Measure");
```

```
close();
selectWindow("C4-AVG_" + original);
close();
```

SUPPLEMENTARY TABLES AND FIGURES

Please browse Full text version of this manuscript to see Supplementary Figures, Tables and Datasets.

Supplementary Figures 1-10 and legends are listed below.

Suppl. Figure 1. Microarray based expression analysis of SIRT isoforms in MCF7 cells at day 4 of doxycycline-induced TACC3 shRNA expression (A) or four days upon γ-irradiation (γIR) using 20 Gy as single dose (B). For SIRT3 and SIRT5, three oligos each were detected on the original arrays with rather inconsistent expression changes. Only SIRT4 mRNA levels were significantly and consistently upregulated both in TACC3-depleted and γ-irradiated cells. Mean ± s.d. from four replicates of TACC3-depleted or γ-irradiated cells *vs*. controls. To evaluate statistical significance, Student's t-tests were performed (*p<0.05).

Suppl. Figure 2. Imaging-based analysis of the expression and colocalization of SIRT4 with the mitochondrial marker MTCO2 in γ-irradiated human dermal fibroblasts. Confocal sections (0.5 μm) depicting MTCO2 (green) – SIRT4 (red) and their colocalization (yellow to orange) are shown for control (sham treated) and γ-irradiated fibroblasts.

Suppl. Figure 3. qPCR-based quantification of selected and senescence-associated miRNAs in MCF7 cells at day 4 of doxycycline-induced TACC3 shRNA expression. (**A**) members of the miR-17-92 and miR-106a-363 clusters are downregulated upon TACC3 depletion. (**B**) qPCR-based expression analysis of miRNAs which were differentially expressed upon TACC3 depletion (suppl. Table 1). Means ± s.d. from three independent experiments analysing TACC3-depleted cells *vs*. controls are depicted. To evaluate statistical significances, Student's t-tests were performed (*p<0.05; **p<0.01).

Suppl. Figure 4. Prediction of miRNA binding sites using the TargetScan software. (**A**) Overview of candidate miRNAs binding to the 3'-untranslated region (3'-UTR) of the *SIRT4* gene in vertebrates. The putative binding site for miR-15/16/195/424/497 (all miRNAs display the same seed region of 8 bp) is highly conserved among vertebrates (Hsa, *Homo sapiens*). Among these miRNAs, only miR-15b was downregulated in TACC3-depleted MCF7 cells undergoing premature senescence (suppl. Table 1). (**B**) Comparison between conserved and poorly conserved miRNA sites in the human *SIRT4* gene. Algorithms used by TargetScan (www.targetscan.org) have been described elsewhere [5-7].

Suppl. Figure 5. Differentiation between extrinsic (UV radiation) and intrinsic (chronological) skin aging through determination of the SCINEXA™ score. Human skin samples obtained from neck *vs.* buttock skin of the same individuals (age ranges: 18-25 years and 60-66 years; median ± SEM) were assessed for multiple parameters as previously described [4] in order to distinguish between extrinsic (score >2) and intrinsic (score <2) skin aging. To evaluate statistical significance, Wilcoxon signed rank tests were performed (***p<0.001).

Suppl. Figure 6. Quantitative analysis of members of the miR-15/16 family in primary human dermal fibroblasts. Determination of copy numbers of the indicated miRNAs of the miR-15/16 family in fibroblasts four days (**A**) and six days (**B**) after γIR (20 Gy) as compared to control (sham treated) cells. Means ± s.d. from six independent experiments are depicted. To evaluate statistical significance for each miRNA, ANOVA (or on ranks) SNK was performed. For clarity, results are depicted for each day separately and sorted by copy number ranking (*p<0.05).

Suppl. Figure 7. Increased expression of SMC4 and DLEU2 in human photoaged skin. Analysis of SMC4 and DLEU2 expression by real-time quantitative RT-PCR in human skin samples obtained from neck *vs.* buttock skin of the same individuals [age ranges: 18-25 years (**A**) and 60-66 years (**B**); n=13-14 per group; median ± SEM]. To evaluate statistical significance, Wilcoxon signed rank tests were performed (***p<0.001).

Suppl. Figure 8. The protein levels of E2F1, a putative regulator of the miR-15/16 – SIRT4 axis, decrease during stress induced senescence. E2F1 protein levels were analysed in MCF7 cells at day 6 upon doxycycline-induced TACC3 shRNA expression (**A**) or γ-irradiation (γIR) using 20 Gy as single dosis (**B**).

Suppl. Figure 9. Suppression of senescence-associated SIRT4 upregulation in MCF7 cells by oligonucleotides mimicking the function of endogenous miR-15b. (**A**) Extrinsically applied miR-15b mimics inhibit basal SIRT4 mRNA levels and prevent their upregulation in MCF7 cells driven into premature senescence through TACC3 depletion (n=3

independent experiments, mean ± s.d.). To evaluate statistical significance, ANOVA on ranks SNK were performed (*p<0.05). (**B**) The 3'-UTR of the human SIRT4 mRNA is a direct target of miR-15b. HeLa cells were co-transfected with firefly luciferase reporter plasmids harbouring the SIRT4 - 3'-UTR or a mutated SIRT4 - 3'-UTR (mut.) and the pRL-SV40 renilla luciferase vector together with oligonucleotides mimicking endogenous miR-15b function ("miR-15b mimics") or control oligonucleotides. Results are given as means ± s.d (n=4 independent experiments with all transfections performed in triplicate). To evaluate statistical significance, ANOVA on Ranks SNK was performed (*p<0.05).

Suppl. Figure 10. Transfection of MicroRNA-15b inhibitors does not result in an inhibition of glutamate dehydrogenase (GDH) activity in human dermal fibroblasts. Fibroblasts were transfected with miR-15b inhibitors or control oligonucleotides and GDH activity was determined at the indicated days following. N=5-6, mean ± s.d.; to evaluate statistical significances, Mann-Whitney rank sum test were performed.

SUPPLEMENTARY FERERENCES

1. Schmidt S, Schneider L, Essmann F, Cirstea IC, Kuck F, Kletke A, Jänicke RU, Wiek C, Hanenberg H, Ahmadian MR, Schulze-Osthoff K, Nürnberg B and Piekorz RP. The centrosomal protein TACC3 controls paclitaxel sensitivity by modulating a premature senescence program. Oncogene. 2010; 29:6184-6192.

2. Schneider L, Essmann F, Kletke A, Rio P, Hanenberg H, Schulze-Osthoff K, Nürnberg B and Piekorz RP. TACC3 depletion sensitizes to paclitaxel-induced cell death and overrides p21WAF-mediated cell cycle arrest. Oncogene. 2008; 27:116-125.

3. Schneider L, Essmann F, Kletke A, Rio P, Hanenberg H, Wetzel W, Schulze-Osthoff K, Nürnberg B and Piekorz RP. The transforming acidic coiled coil 3 protein is essential for spindle-dependent chromosome alignment and mitotic survival. J Biol Chem. 2007; 282:29273-29283.

4. Vierkötter A, Ranft U, Kramer U, Sugiri D, Reimann V and Krutmann J. The SCINEXA: a novel, validated score to simultaneously assess and differentiate between intrinsic and extrinsic skin ageing. J Dermatol Sci. 2009; 53:207-211.

5. Friedman RC, Farh KK, Burge CB and Bartel DP. Most mammalian mRNAs are conserved targets of microRNAs. Genome Res. 2009; 19:92-105.

6. Grimson A, Farh KK, Johnston WK, Garrett-Engele P, Lim LP and Bartel DP. MicroRNA targeting specificity in mammals: determinants beyond seed pairing. Mol Cell. 2007; 27:91-105.

7. Lewis BP, Burge CB and Bartel DP. Conserved seed pairing, often flanked by adenosines, indicates that thousands of human genes are microRNA targets. Cell. 2005; 120:15-20.
