# Supplementary material for: MicroRNA-15b regulates mitochondrial ROS production and the senescence-associated secretory phenotype through sirtuin 4/SIRT4
Source: Aging (Albany NY). 2016 Feb 26;8(3):484–505. doi: 10.18632/aging.100905 (PMC4833141; doi:10.18632/aging.100905)
Supplement: Supplementary file 2 [file aging-08-484-s002.pdf]

Suppl. Table 1: miRNAs differentially regulated upon TACC3 depletion in MCF7 cells thereby undergoing premature senescence.

miRNA	Regulation	Fold increase / decrease	P value
hsa-miR-146b-5p	up	3,82	0,0094
hsa-miR-1977	up	2,34	0,0345
hsa-miR-181a*	up	2,20	0,0174
hsa-miR-193b*	up	2,14	0,0243
hsa-miR-141	up	1,98	0,0046
hsa-miR-1973	up	1,92	0,0229
hsa-miR-29b-1*	up	1,77	0,0033
hsa-miR-125b	up	1,72	0,0322
hsa-miR-21*	up	1,71	0,0213
hsa-miR-491-3p	up	1,69	0,0001
hsa-miR-30c	up	1,68	0,0203
hsa-miR-200a*	up	1,64	0,0189
hsa-miR-30e	up	1,54	0,0450
hsa-miR-200c*	up	1,53	0,0424
hsa-miR-1275	up	1,53	0,0002
hsa-miR-183*	down	3,34	0,0037
hsa-miR-501-3p	down	2,65	0,0046
hsa-miR-345	down	2,63	0,0140
hsa-miR-18a	down	2,27	0,0071
hsa-miR-194	down	2,07	0,0429
hsa-miR-33a	down	2,06	0,0005
hsa-miR-218	down	1,99	0,0341
hsa-miR-32	down	1,94	0,0046
hsa-miR-17	down	1,93	0,0205
hsa-miR-18b	down	1,84	0,0169
hsa-miR-19a	down	1,83	0,0115
hsa-miR-19b	down	1,83	0,0109
hsa-miR-7	down	1,82	0,0388
hsa-miR-192	down	1,77	0,0072
hsa-miR-19b-1*	down	1,75	0,0011
hsa-miR-101	down	1,70	0,0301
hsa-miR-362-3p	down	1,70	0,0051
hsa-miR-769-5p	down	1,64	0,0164
hsa-miR-20a	down	1,62	0,0339
hsa-miR-15b	down	1,61	0,0207
hsa-miR-590-5p	down	1,57	0,0243
hsa-miR-503	down	1,54	0,0283
hsa-miR-219-5p	down	1,52	0,0284

Analysis of differentially expressed miRNAs using the Genespring software (ver11) (expression changes ≥ 1.5-fold were determined four days after DOX-induced TACC3 shRNA expression that triggers premature senescence; p≤0.05; student‘s t-test).

Suppl. Table 2: Overview of the number of differentially regulated, putative miRNA target genes *vs*. the total number of candidate miRNA target genes.

	miRNA	Differentially regulated putative target genes	Candidate target genes (total)	P value
**1**	**hsa-miR-17**	**138**	**1601**	**0,000011**
**2**	**hsa-miR-20a**	**138**	**1594**	**0,000008**
**3**	**hsa-miR-30e**	**129**	**1445**	**0,000004**
**4**	**hsa-miR-30c**	**124**	**1440**	**0,000031**
**5**	**hsa-miR-19a**	**124**	**1385**	**0,000004**
**6**	**hsa-miR-19b**	**124**	**1383**	**0,000004**
**7**	***hsa-miR-15b***	***108***	***1359***	***0,001533***
8	hsa-miR-21*	99	1345	0,021258
**9**	**hsa-miR-32**	**96**	**992**	**0,000002**
10	hsa-miR-218	75	928	0,005707
11	hsa-miR-101	65	855	0,032452
12	hsa-miR-125b	61	845	0,088203
**13**	**hsa-miR-7**	**53**	**473**	**0,000007**
14	hsa-miR-503	35	355	0,002857
15	hsa-miR-18a	32	320	0,003698
16	hsa-miR-18b	32	320	0,003698
17	hsa-miR-590-5p	30	307	0,006633
18	hsa-miR-219-5p	29	417	0,342896
19	hsa-miR-491-3p	25	278	0,028984
20	hsa-miR-194	23	363	0,651865
21	hsa-miR-362-3p	21	327	0,634296
22	hsa-miR-146b-5p	20	168	0,002443
23	hsa-miR-33a	15	376	0,148934
24	hsa-miR-192	13	125	0,051686
25	hsa-miR-345	11	140	0,277760
26	hsa-miR-501-3p	9	241	0,211704
27	hsa-miR-769-5p	7	126	1,000000
28	hsa-miR-1275	3	90	0,493812

Analysis of putative target mRNAs differentially regulated by miRNAs was performed using the Genespring software (ver11). Expression changes ≥ 1.75 were determined four days after DOX-induced TACC3 shRNA expression and senescence induction ($p \leq 0.05$; student's t-test). As indicated in the last column, significant correlations between the total number of putative target mRNAs *vs.* the number of differentially expressed candidate target mRNAs were determined using Fisher's exact test ($p \leq 0.05$) and a Bonferroni correction (highly significant miRNA-mRNA correlations with $p \leq 0.0018$ are indicated in bold).

A

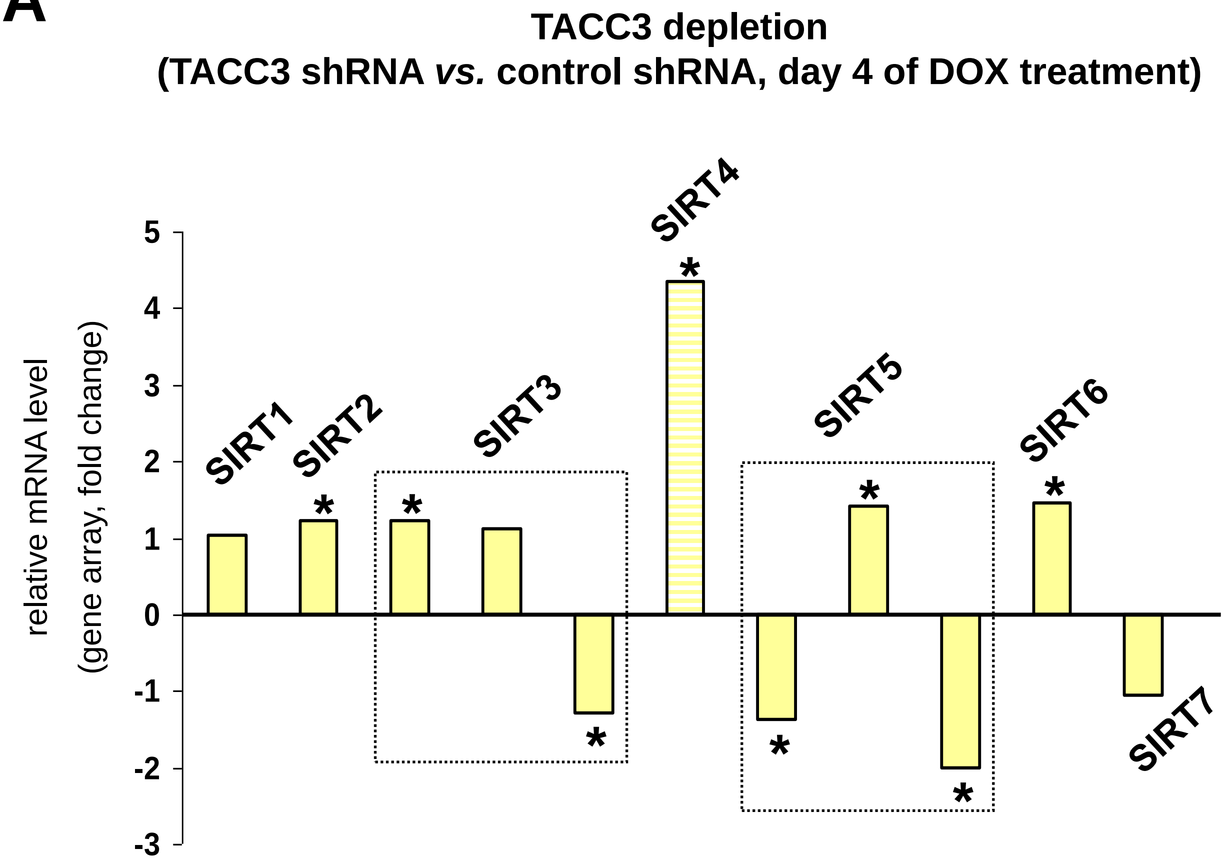

B

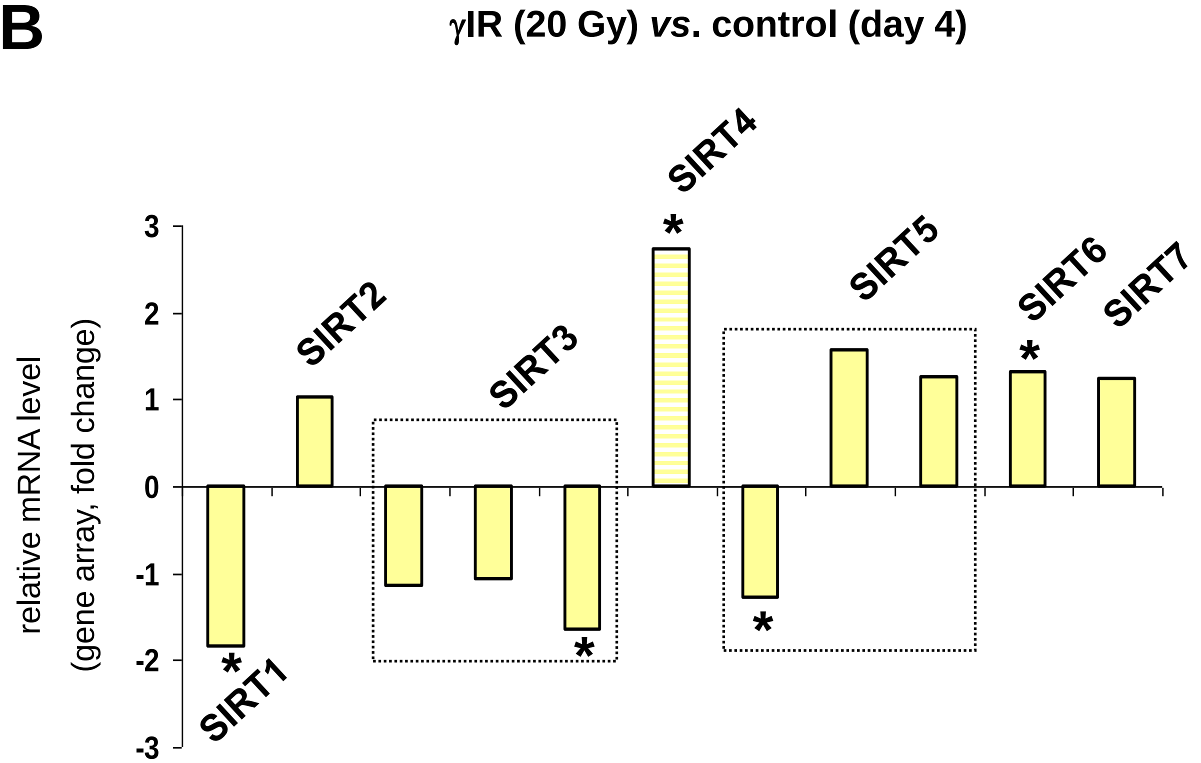

suppl. Figure 1

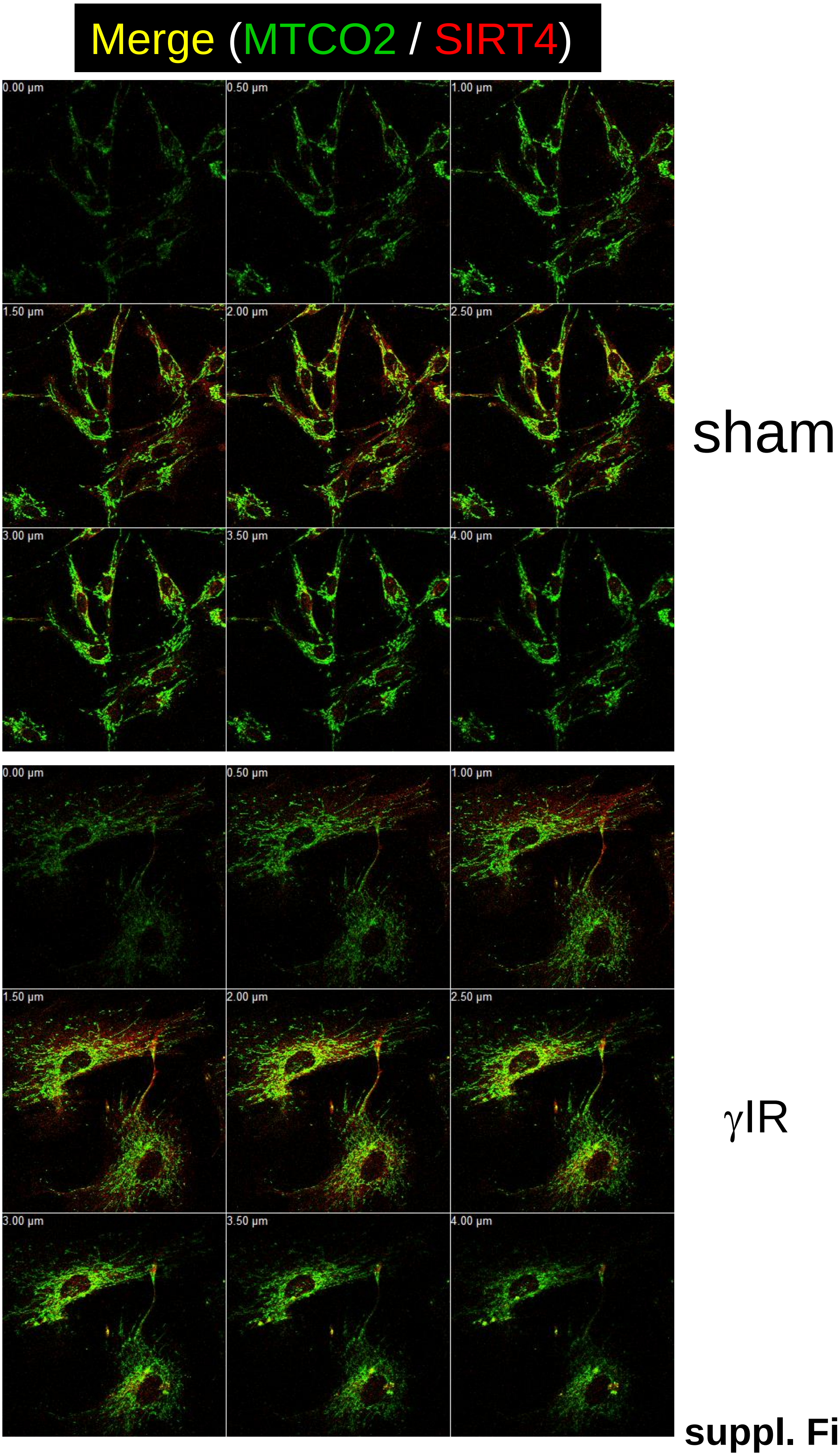

suppl. Figure 2

A

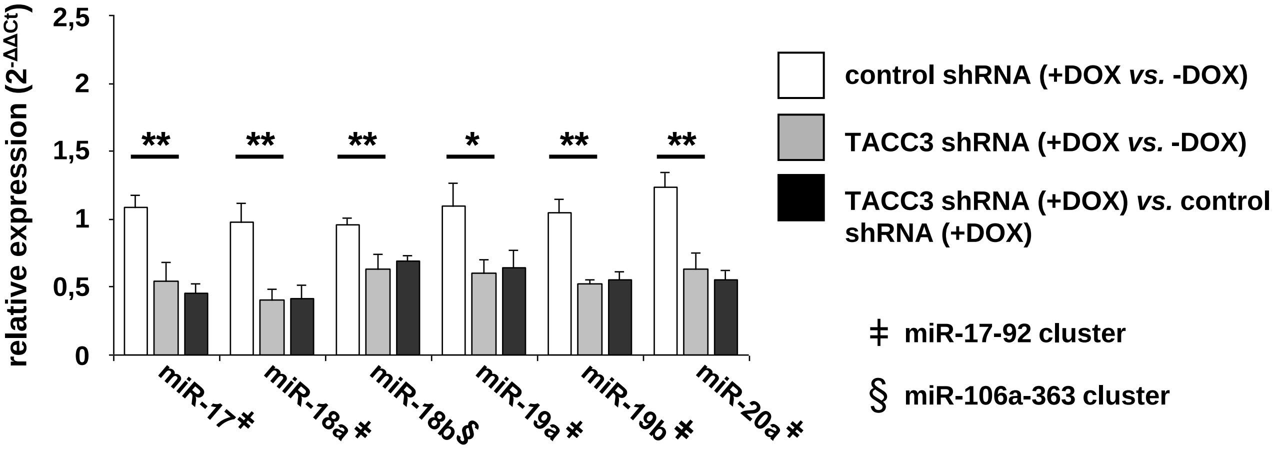

B

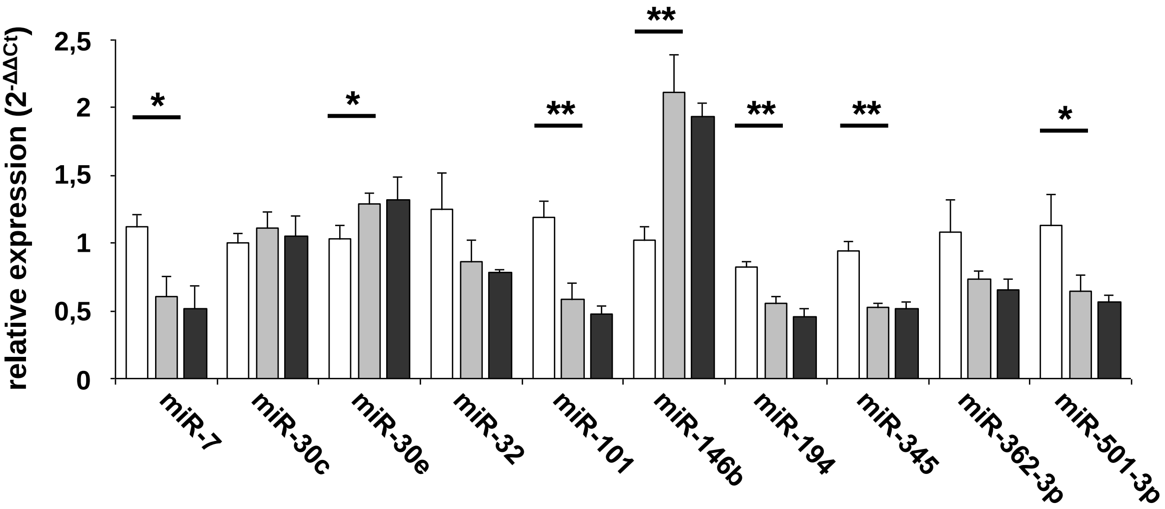

suppl. Figure 3

A

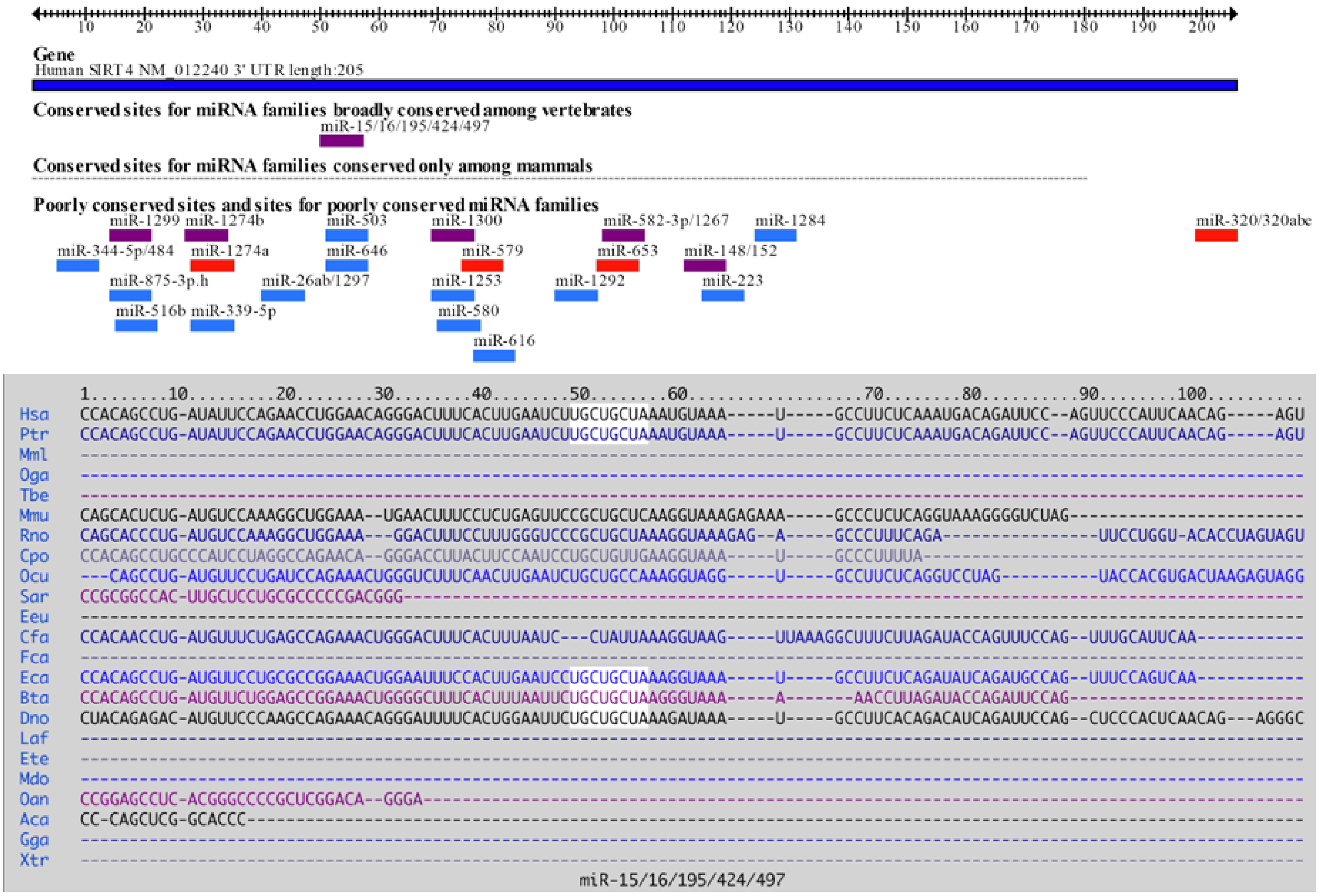

http://www.targetscan.org/cgi-bin/vert_50/view_gene.cgi?gs=SIRT4&taxid=9606&members=&showcnc=1&shownc=1

B

Human SIRT4 3' UTR miRNA Table

Table sorted by total context score [Sort table by aggregate P_{CT}]

miRNA families broadly conserved among vertebrates

miRNA	conserved sites				poorly conserved sites				Total Context score	Aggregate P_{CT}
	Total	8mer	7mer-m8	7mer-1A	Total	8mer	7mer-m8	7mer-1A		
miR-15/16/195/424/497	1	1	0	0	0	0	0	0	-0.47	< 0.1
miR-148/152	0	0	0	0	1	1	0	0	-0.32	0.22
miR-503	0	0	0	0	1	0	0	1	-0.20	< 0.1
miR-26ab/1297	0	0	0	0	1	0	0	1	-0.16	< 0.1
miR-223	0	0	0	0	1	0	0	1	-0.13	0.20

http://www.targetscan.org/cgi-bin/vert_50/view_genetable.cgi?gs=SIRT4&taxid=9606&members=&showcnc=1&shownc=1&sortText=cs

suppl. Figure 4

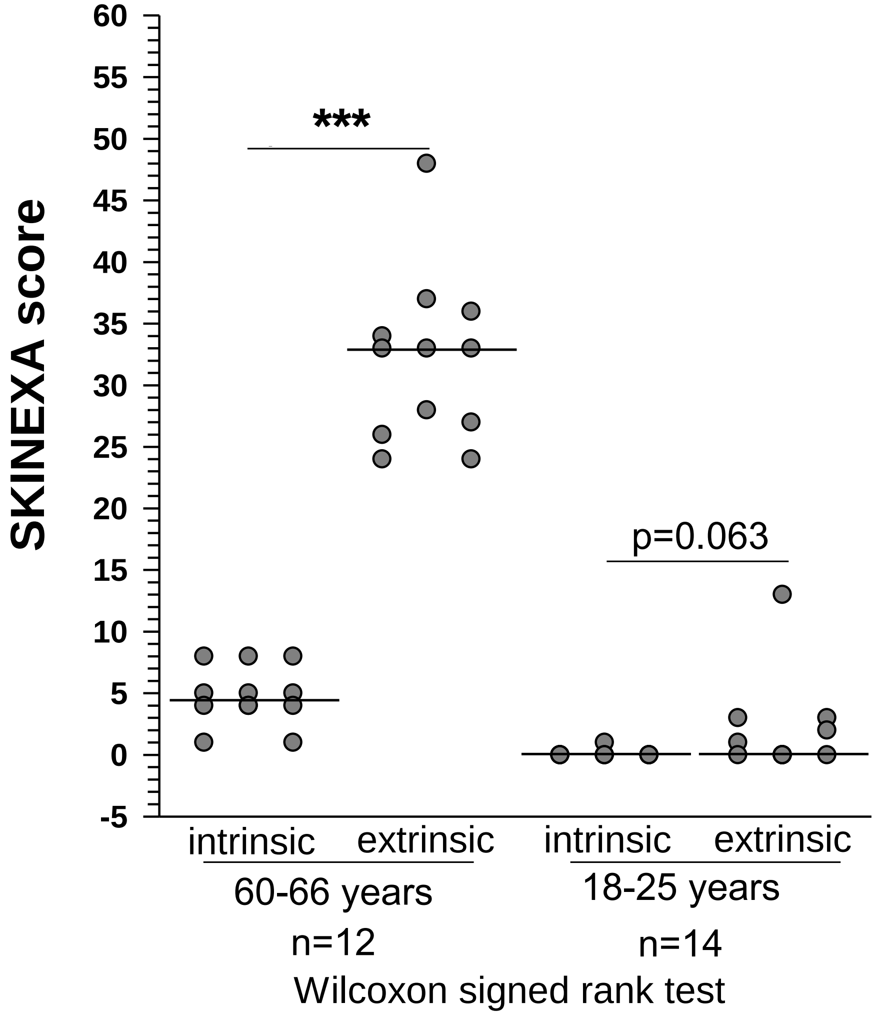

suppl. Figure 5

A

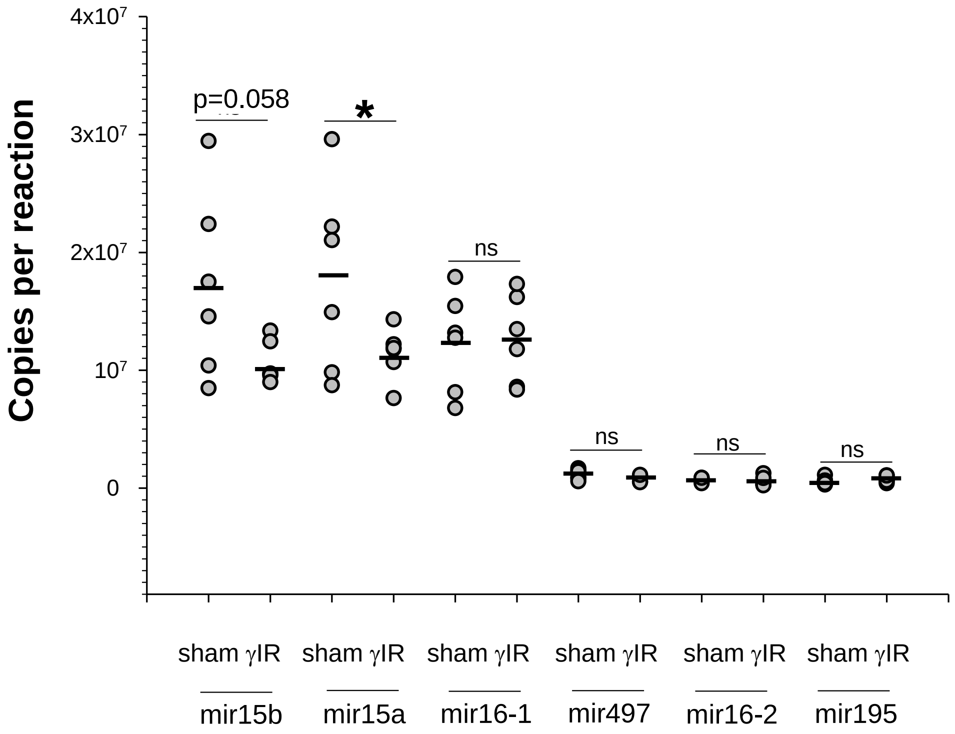

B

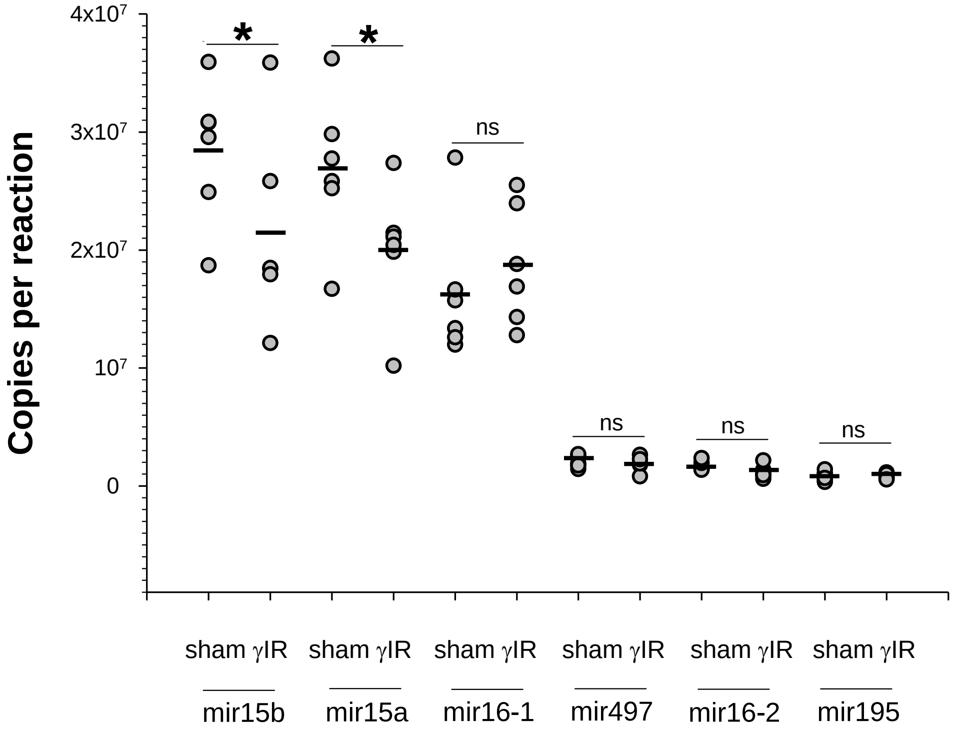

suppl. Figure 6

A

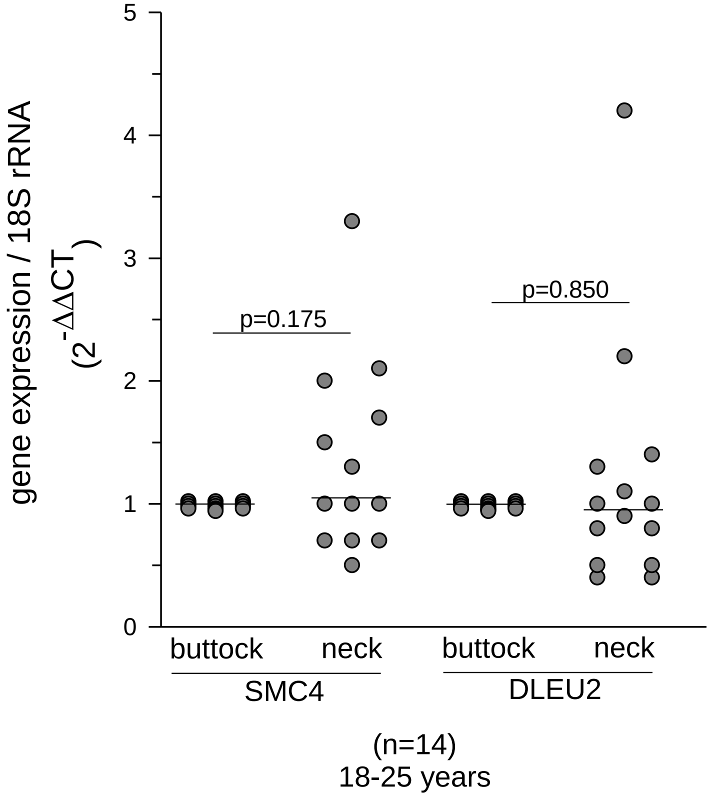

B

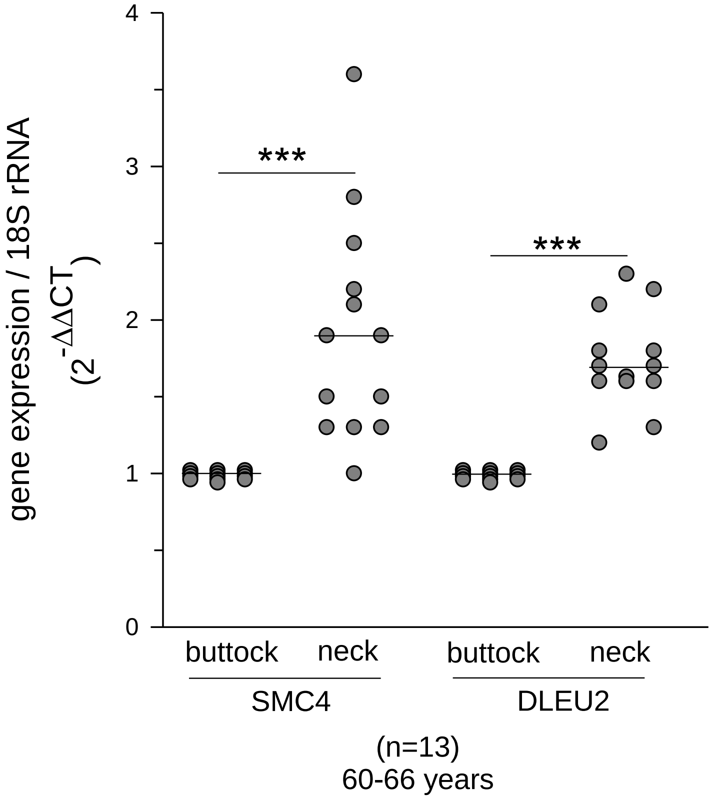

suppl. Figure 7

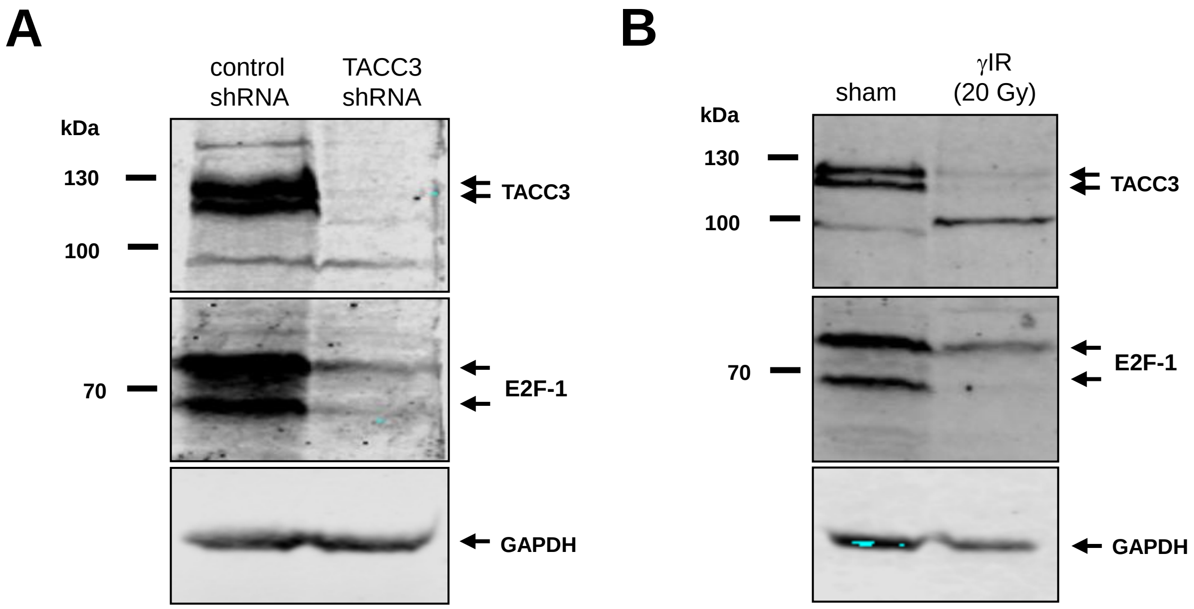

suppl. Figure 8

A

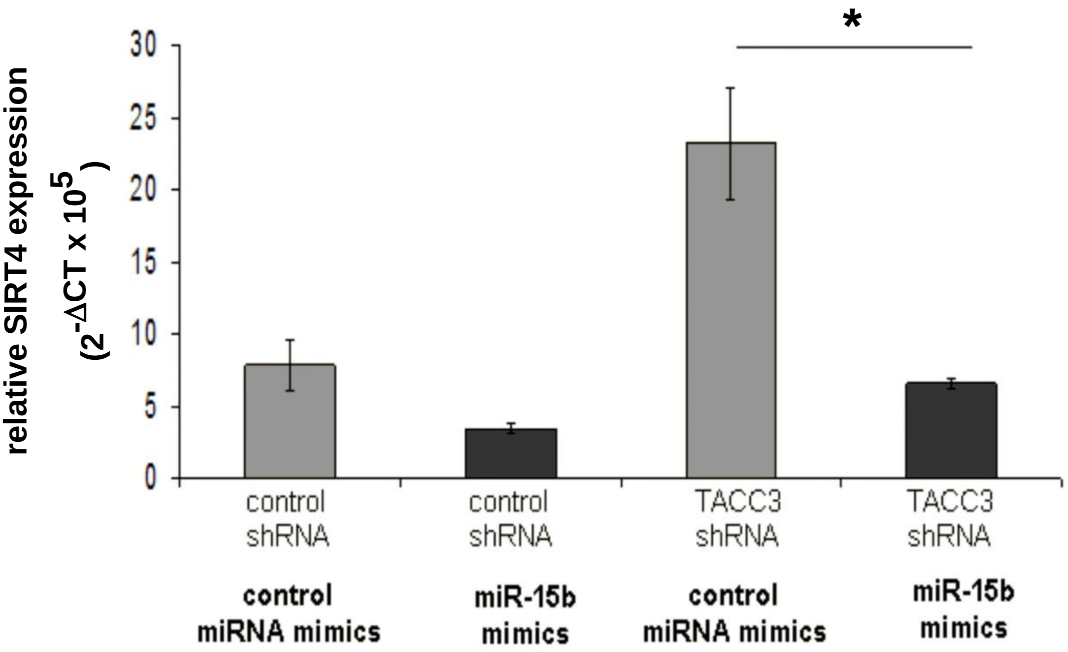

B

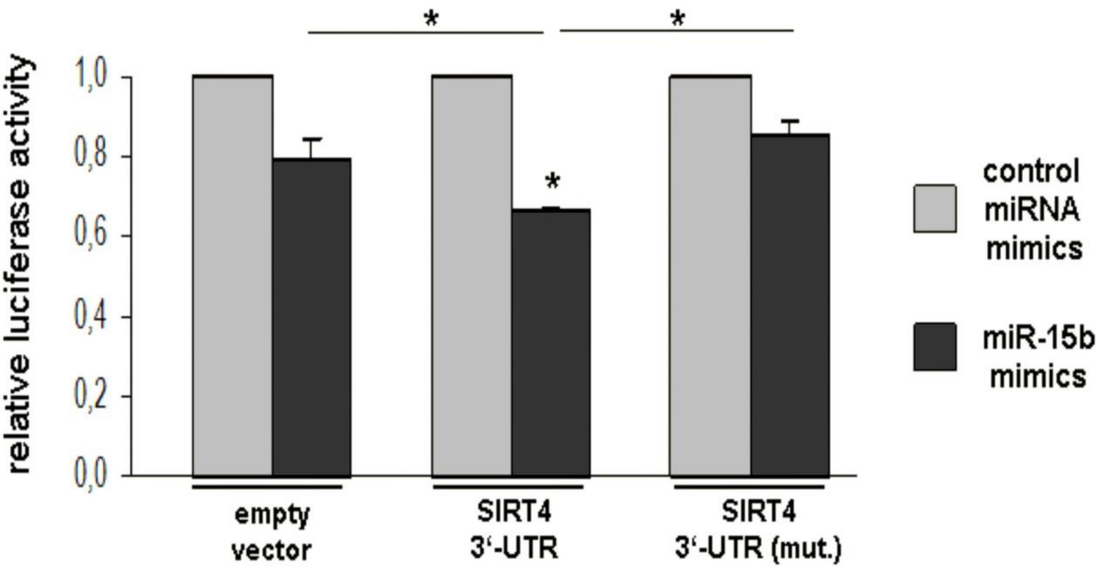

suppl. Figure 9

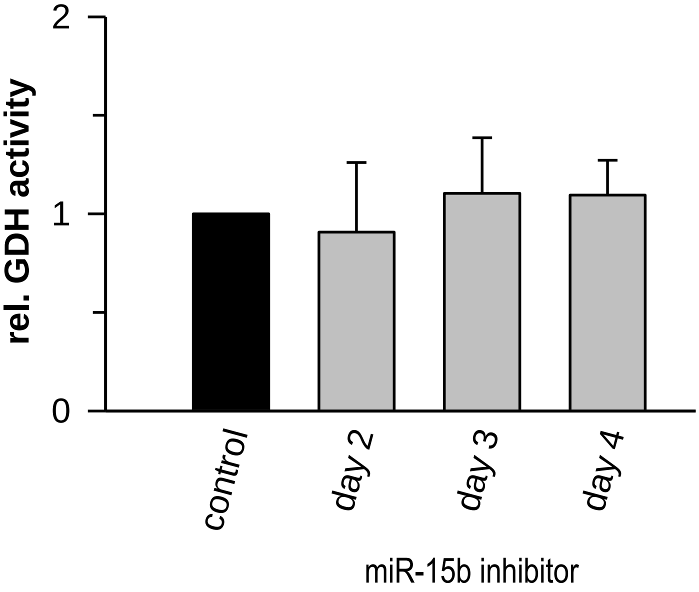

suppl. Figure 10
