# Supplementary material for: MicroRNA-15b regulates mitochondrial ROS production and the senescence-associated secretory phenotype through sirtuin 4/SIRT4
Source: Aging (Albany NY). 2016 Feb 26;8(3):484–505. doi: 10.18632/aging.100905 (PMC4833141; doi:10.18632/aging.100905)
Supplement: Supplementary file 3 [file aging-08-484-s003.pdf]

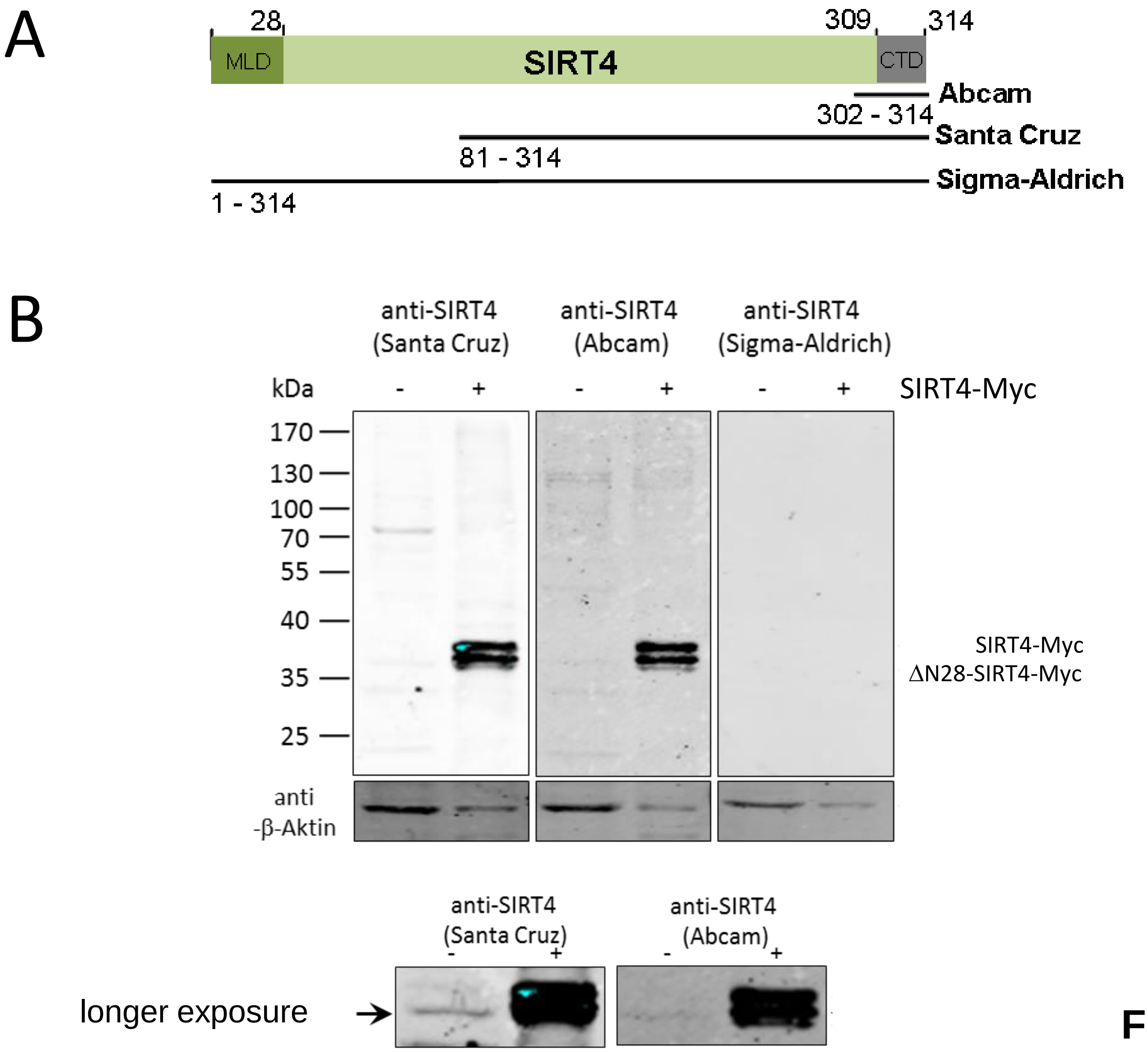

Figure 1
(for referee's use only)

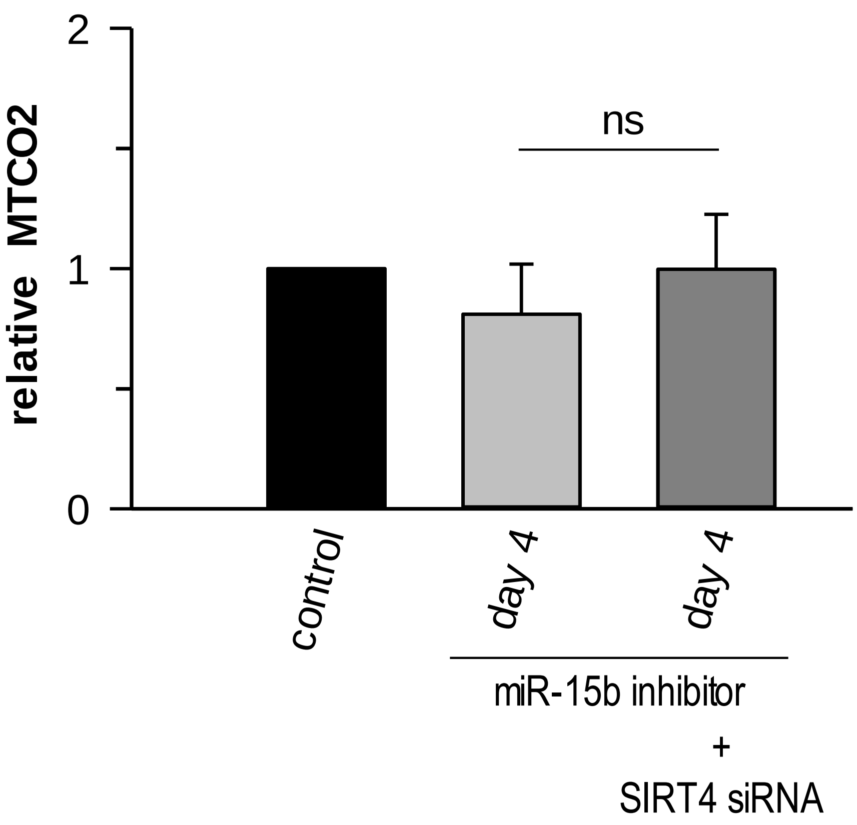

Figure 2
(for referee‘s use only)

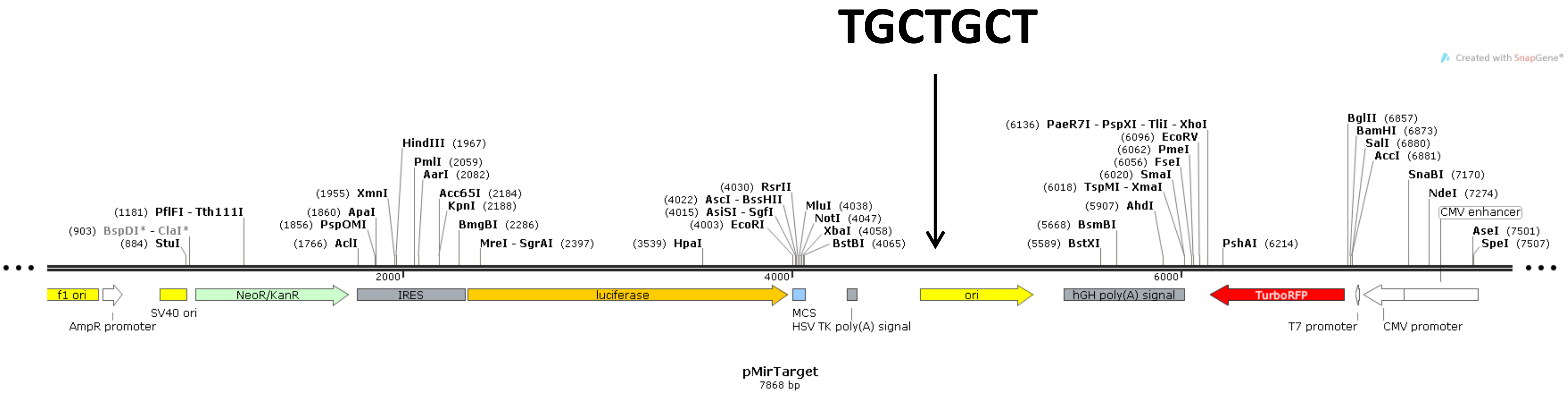

Complete seed seq. for miR-15b: ***5'-TGCTGCTA-3'***

Figure 3
(for referee's use only)
